# SLC4A7 Drives NSCLC Progression and Immune Evasion via pH Dysregulation: Its Targeting Synergizes with Anti-PD-1/L1 Therapy

**DOI:** 10.7150/ijbs.129129

**Published:** 2026-03-30

**Authors:** Hao Qin, Dongfang Tang, Zheng Li, Fuzhi Yang, Jing Wang, Xiucheng Liu, Chang Chen, Wen Gao

**Affiliations:** 1 Department of Thoracic Surgery, Huadong Hospital, Fudan University, Shanghai, 200041, China.; 2 Department of Thoracic Surgery, Shanghai Pulmonary Hospital, Tongji University School of Medicine, Shanghai, 200433, China.

**Keywords:** non-small cell lung cancer, tumor microenvironment, bicarbonate transporter, immunotherapy

## Abstract

Solid tumors create an acidic tumor microenvironment (TME) that drives cancer progression, therapy resistance, and immune evasion. Bicarbonate is crucial for maintaining acid-base balance, however, its role in non-small cell lung cancer (NSCLC) remains unclear. Through the analysis of single-cell RNA sequencing data and the TCGA and CPTAC databases, we identified solute carrier family 4 member 7 (SLC4A7) as the predominantly expressed bicarbonate transporter in NSCLC. Functionally, SLC4A7 knockdown impaired bicarbonate uptake, resulting in intracellular acidification and extracellular alkalinization. This phenomenon led to a decrease in glycolysis and subsequently suppressed the growth and metastasis of NSCLC. Both *in vivo* and *in vitro* data demonstrate that the alkalinization of the TME induced by Slc4a7 knockout enhances the infiltration and function of cytotoxic T cells, significantly inhibiting tumor growth. Additionally, Slc4a7 knockout exhibits synergistic antitumor efficacy in combination with PD-1/PD-L1 immune checkpoint inhibitors. Mechanistically, integrative analysis of RNA-seq and ATAC-seq data identified CTCF as a transcription factor regulating SLC4A7 expression. In summary, our study demonstrates that SLC4A7-mediated bicarbonate transport is crucial for maintaining acid-base homeostasis in NSCLC and represents a promising therapeutic target for this disease.

## Introduction

The acidic tumor microenvironment, a hallmark of all solid tumors, plays a crucial role in tumor progression^1^, ^2^. To prevent intracellular acidosis, tumor cells adeptly maintain an acidic extracellular pH (pHe) while preserving a relatively alkaline intracellular pH (pHi)^2^-^4^. This specialized acid-base microenvironment in cancer tissues promotes tumor progression, therapeutic resistance, and immune evasion^4^. Therefore, disrupting the unique pH gradient within tumor cells has emerged as a promising strategy for cancer therapy.

Highly proliferative tumors predominantly rely on glycolysis for energy production, a phenomenon known as the Warburg effect^5^, ^6^. This effect is a well-established adaptive feature of cancer cells and other rapidly proliferating cells^7^. Enhanced glycolysis in tumor cells leads to excessive production of H+ (including lactate and carbonic acid as metabolic end products), which accumulate intracellularly, thereby adversely affecting metabolic activity and potentially limiting the malignant behavior of cancer cells^4^, ^8^-^10^. To prevent intracellular acidification, cancer cells have developed complex mechanisms for net acid extrusion, including Na^+^/HCO₃⁻ cotransporters, Na⁺/H⁺ exchangers, H⁺-ATPases, and monocarboxylate transporters^11^, ^12^. These adaptations are essential for maintaining metabolic activity and supporting malignant functions. Recent studies have shown that the disruption of net acid extrusion mechanisms can significantly inhibit tumor growth, metastasis, and glycolysis^13^-^15^. The cellular transport of bicarbonate (HCO₃⁻) is crucial for maintaining acid-base homeostasis both intracellularly and extracellularly, a process that is mediated by various bicarbonate transporters^16^. However, the function and underlying mechanism of bicarbonate transporters in NSCLC remain unclear. Bicarbonate transporters are classified into two families: SLC4 and SLC26^11^. The SLC4 family comprises 10 genes, five of which (SLC4A4, SLC4A5, SLC4A7, SLC4A8, and SLC4A10) encode Na⁺-coupled bicarbonate transporters that facilitate bicarbonate import across the plasma membrane^11^, ^16^. Notably, SLC4A7 encodes the electroneutral Na⁺/HCO₃⁻ cotransporter (NBCn1), which is critical for regulating both intracellular and extracellular acid-base balance^16^. Studies in breast cancer, pancreatic ductal carcinoma, and head and neck squamous cell carcinoma have demonstrated that inhibition of SLC4A7 can suppress tumor progression^12^, ^17^-^20^. However, the specific role of SLC4A7 in regulating tumor pH in lung cancer remains largely unexplored.

The tumor microenvironment (TME) represents a highly complex biological system, wherein immune cells are pivotal functional components^21^-^23^. Recent evidence indicates that the acidic microenvironment not only impacts tumor cells directly but also alters the composition and function of immune cells within the TME^4^, ^24^-^28^. Furthermore, tumor acidity has been shown to directly compromise the efficacy of immune checkpoint inhibitors^29^, ^30^. Notably, acidity impairs the antitumor response of tumor-infiltrating immune cells, thereby facilitating immune evasion^31^-^34^. Under acidic conditions, the functionality of T cells and natural killer cells is diminished, whereas immunosuppressive myeloid cells and regulatory T (Treg) cells are expanded^27^, ^35^-^37^. Recent studies have demonstrated that inhibition of SLC4A4 activity in pancreatic cancer cells can restore T cell function and attenuate macrophage-mediated immunosuppression^38^. Therefore, targeting pH regulators to prevent tumor acidification represents a promising therapeutic strategy in the context of antitumor immunity and immunotherapy.

In this study, our *in vitro* and *in vivo* experiments demonstrate that SLC4A7-mediated bicarbonate transport plays a crucial role in maintaining acid-base homeostasis in NSCLC. The knockdown of SLC4A7 not only inhibits tumor cell growth and metastasis but also enhances the infiltration and activation of CD8+ T cells within tumors, thereby increasing the efficacy of PD-1/PD-L1 checkpoint inhibitors.

## Methods and Materials

### Cell line

Human non-small cell lung cancer cell NCI-H1299, LUAD cell line A549 and LUSC cell line NCI-H226, NCI-H1703 were purchased from Procell Life Science & Technology (Wuhan, China). All cell lines were rigorously verified using genomic short tandem repeat (STR) profiling analysis. H1299, H226 and H1703 cell were cultured in RPMI-1640 medium supplemented with 10% fetal bovine serum (Sigma) and 1% Penicillin-Streptomycin Solution, A549 cell was cultured in DMEM medium supplemented with 10% fetal bovine serum (FBS, Sigma) and 1% Penicillin-Streptomycin (PS). All cells were cultured in a humidified incubator containing 5% carbon dioxide. The mouse cancer cell lines: KP cells were generated from Kras^G12D^, Trp53^-/-^ C57BL/6 mouse and cultured in RPMI-1640 supplemented with 10% FBS and 1% P/S.

### Mouse

Balb/c nude mouse (6-8 weeks, male) and Severe immunodeficiency NCG (NOD/ShiLtJGpt-Prkdc^em26Cd52^Il2rg^em26Cd22^/Gpt) male mouse were purchased from GemPharmatech LLC (Nanjing, China), C57BL/6 mouse (6-8 weeks, male) were purchased from Beijing Vital River Laboratory Animal Technology Co., Ltd (Beijing, China). All animal experiments were approved by the Animal Care and Use Committee of the Laboratory Animal Center of Fudan University and complied with the NIH Guide for the Care and Use of Laboratory Animals. The IACUC number is 202501FD0004.

### *In vivo* experiments

A total of 6 × 10^6 A549 cells and 8 × 10^6 H226 cells in 200 μL were subcutaneously injected into the axillary region of Balb/c nude mice. Tumor growth was monitored weekly or at the experimental endpoint by measuring tumor volume according to the formula: length × width²/2. For the tail vein metastasis assay, 5 × 10^6 A549 cells and 6 × 10^6 H226 cells in 100 μL were injected into Balb/c nude mice via the tail vein. At the endpoint, mice were euthanized by cervical dislocation, and lung tissues were collected for histological analysis.

For orthotopic and subcutaneous models, 5 × 10^5 KP cells in 100 μL were injected via the tail vein and subcutaneously into C57BL/6 mice to establish orthotopic carcinoma and subcutaneous tumor models, respectively. Mice were sacrificed at humane endpoints. Immunotherapy was administered by intraperitoneal injection of control IgG or anti-PD-1/PD-L1 antibody (10 mg/kg body weight) three times per week. For *in vivo* depletion of CD8+ T cells, mice received intraperitoneal injections of anti-CD8 antibody (10 mg/kg) three days prior to tumor inoculation and twice weekly thereafter. For *in vivo* inhibition of SLC4A7, mice were administered DIDS (15 mg/kg) by intraperitoneal injection once daily.

### Immunohistochemical (IHC) staining

Paraffin-embedded tissue sections were deparaffinized and subjected to antigen retrieval. After natural cooling, slides were placed in PBS and washed three times on a decolorizing shaker for 5 minutes each. After washing, sections were incubated in 3% hydrogen peroxide solution at room temperature in the dark for 25 minutes to block endogenous peroxidase activity. Sections were then covered with 3% bovine serum albumin (BSA) within a hydrophobic barrier to block nonspecific binding and incubated at room temperature for 30 minutes. After removing the blocking solution, 1:200 diluted primary antibody against SLC4A7 was added and the sections were incubated overnight at 4°C. The next day, sections were washed to remove the primary antibody and incubated with the appropriate secondary antibody at room temperature for 60 minutes. After completion of antibody incubation, DAB substrate solution was applied for color development. Sections were counterstained with hematoxylin for 3 minutes. Slides were mounted and examined under a microscope for evaluation.

Results were independently evaluated by two pathologists. Immunoreactivity intensity was scored on a scale of 0 to 3 (0, negative; 1, weak; 2, moderate; 3, strong), and the percentage of positively stained cells was graded as follows: 1 (0-25%), 2 (26-50%), 3 (51-75%), and 4 (76-100%). The staining index (0-12) was calculated by multiplying the intensity score by the positive area score.

### Immunofluorescence

Dewaxing, antigen retrieval, and blocking procedures for paraffin-embedded sections were performed as described for immunohistochemistry (IHC). The following primary antibodies were applied at the specified dilutions: SLC4A7 (Thermo Fisher Scientific, 1:200), CD8 (Abcam, 1:200), Pan-CK (Abcam, 1:500), and DAPI (BioLegend, 30 nM). After primary antibody incubation, species-specific fluorescent secondary antibodies were applied. Stained samples were examined and quantified using a fluorescence microscope.

### Lentiviral transductions

The transfection lentivirus was commissioned to Shanghai Genechem Biotechnology Co. for construction. Cells were seeded in a 6-well plate 24 hours before transfection, and the lentivirus was added to the six-well plate to be transfected at the recommended Moi value (MOI = 10) the next day. After 12 hours of transfection, the culture medium was replaced with puromycin for screening for one week for subsequent experiments. For Slc4a7 knockout in KP cells, slowly thaw the sgSlc4a7 RNP-vector complex and add 10 μL per well in multiple doses, slowly adding the mixture and shaking thoroughly. 48 hours after transfection, determine knockout efficiency by western blot.

### Transwell assay

The Tranwell experiment is divided into two parts: invasion and migration. For the invasion experiment, the 8μm chamber was infiltrated with serum-free culture medium before the experiment began. Subsequently, a 1:5 diluted matrix gel was added to the inside of the chamber, and then placed in a 37-degree incubator for four hours to allow the matrix gel to solidify. After the matrix gel solidified, 800μl of serum-containing culture medium was added to the 24-well plate, and 200μl of serum-free cell suspension was added to the upper chamber. After 24 hours, ice methanol was used for fixation for 15 minutes, crystal violet staining was used for 15 minutes, and then ultrapure water was used to wash and wipe off the cells that did not pass through the upper chamber. Use a Leica microscope to take pictures and count. Migration experiments do not require the laying of matrix gel, and the other steps are the same as the invasion experiment.

### Cell proliferation assays

Cell proliferation was detected using the Cell counting kit-8 (CCK-8) assay, colony formation assay and EdU assay. Cell proliferation ability was detected using CCK8, EDU and clone formation. For the CCK8 experiment, the cells were planted in the same number in a 96-well plate according to the grouping. After the cells adhered to the wall, the supernatant was discarded, and the CCK8 enhancement solution and culture medium mixture were added. The cells were incubated in a 37-degree incubator for 1.5 hours, and then the absorption light was detected at 450nm on the microplate reader, which was recorded as day 0. Then the detection was performed once on the first, second and third days. The cell plating operation of the EDU experiment was the same as above. After 24 hours, the experiment was carried out according to the instructions of the reagent manufacturer. In short, 10μM EDU reagent was added to the culture plate and incubated for 2 hours, followed by staining with Apollo 567 dye. After staining, the cells were observed and photographed under a Leica immunofluorescence microscope. The clone formation was tested using a 6-well cell culture plate. The cells were diluted to 500/ml and added to the six-well plate. After 10 days, the six-well plate was collected, fixed and stained with crystal violet, and the experimental results were counted and statistically analyzed.

### Seahorse experiment

The extracellular acidification rate of cells was detected using the Seahorse assay. The kit was purchased from Agilent, and the experimental steps were carried out according to the steps in the kit, and the drug concentrations were consistent with our previous experiments. In brief, the cells were cultured in a specific cell culture plate the day before the experiment, and the probe was hydrated with sterile ultrapure water. On the day of the experiment, glucose, oligomycin, and FCCP were added in the order indicated in the kit, and then measured on the XFe96 instrument.

### pHi and pHe measurements

pH was determined based on a calibration curve prepared with the same probe dissolved in buffers of different pH. Intracellular pH level was determined with pHrodo Red AM (ThermoFisher, P35372). A549, H1299, H226 and H1703 The control and correspondingly treated A549, H1299, H226 and H1703 were seeded at 8000 cells per well in black-walled, clear-bottomed 96-well plates. Add pHrodoTM dye to cells and incubate at room temperature for 30 minutes. Wash cells twice with pre-warmed Live Cell Imaging Solution. Measure fluorescence using a fluorescence microplate reader (excitation wavelength 555 nm, emission wavelength 590 nm). The pH value was determined based on the calibration curve prepared with the Intracellular pH Calibration Buffer Kit.

pHe was measured directly in the cell culture medium using a single-barrel H+-sensitive microelectrode that was calibrated before and after the measurement with a NaCl solution containing a mixture of KH2PO4 and Na2PO4.

### Bicarbonate uptake

SLC4A7 knockdown and control cells were seeded into 24-well culture plates one day prior to the experiment. On the following day, cells were washed twice with bicarbonate-free medium. Subsequently, cells were incubated in medium supplemented with 5 μCi of [14C] sodium bicarbonate for 5 minutes, followed by lysis in 1 N NaOH. Radioactivity was quantified using liquid scintillation counting.

### *In vivo* [^31^P] magnetic resonance spectroscopy (MRS)

Magnetic resonance spectroscopy (MRS) measurements of size-matched KP subcutaneous tumors were performed using a dedicated 11.7 T small animal MRI system (BioSpec, Bruker BioSpin) following established protocols^39^, ^40^. Animals were anesthetized with inhaled isoflurane, with continuous monitoring of respiratory rate throughout anesthesia. For *in vivo* pH measurement, 800 μL of 245 nM 3-aminopropylphosphonate (3-APP; Sigma-Aldrich) was administered via intraperitoneal injection 30 minutes prior to data acquisition. Animals were positioned in a transmitter/receiver RF coil, and a 1H/31P surface coil (2 cm diameter, Bruker BioSpin) was placed over the tumor site. Tumor regions were selected using T2-weighted, rapid-acquisition, relaxation-enhancement sequences with two distinct slices. Intracellular (pHi) and extracellular (pHe) pH measurements were calculated using jMRUI v5 software, as described in reference 66, based on the chemical shifts between the inorganic phosphate (Pi) and α-ATP peaks in the 31P spectrum for pHi, and between the 3-APP and α-ATP peaks for pHe.

### FACS analysis

Spleen, lung, and subcutaneous tissue samples were mechanically dissociated to generate single-cell suspensions. Red blood cell lysis buffer (BD Pharmingen, 555899) was added to the cell suspension and incubated at room temperature for 10-15 minutes. Cells were centrifuged at 500 × g for 5 minutes, and the supernatant was discarded. The cell pellet was washed and resuspended in 1 mL of 1× PBS to a final concentration of 1 × 10^7 cells/mL. Two microliters of Fc block were added to each sample, followed by incubation at room temperature in the dark for 15 minutes. Cells were then resuspended in 1 mL of 1× PBS. Blank controls, single-positive controls, and fluorescence minus one control were prepared in parallel.

Samples were resuspended in 1 mL of 1× PBS, and 1 μL of viability dye was added. Samples were incubated at room temperature in the dark for 15 minutes, followed by centrifugation at 500 × g for 5 minutes. Cells were washed with 1 mL of Stain Buffer (BD Pharmingen, 554656), centrifuged at 500 × g for 5 minutes, and resuspended in 200 μL of Stain Buffer. The appropriate amount of cell surface fluorescent antibody was added to each tube according to the manufacturer's instructions, followed by incubation at room temperature in the dark for 15 minutes. The specific antibodies are directly labeled as follows: Treg: CD45-APC-Cy7, CD4-FITC, CD25-APC, FOXP3-PE; NK cells: CD45-APC-Cy7, CD3-APC, CD19-FITC, NK1.1-PE-CY7; T cells: CD45-APC-Cy7, CD3-APC, CD4-FITC, CD8-Percy-cy5.5; B cells: CD45-APC-Cy7, CD3-APC-, CD19-FITC; DC cells: CD45-APC-Cy7, CD11c-BV421, MCHII-PE-cy7, CD11b-FITC, CD8-percp-cy5.5; MAC: CD45-APC-Cy7, F4/80-PE-eFluor610, CD11b-FITC, CD86-PE-cy7, CD206-AF647. See Table S1 for the part numbers of related products. Subsequently, 1 mL of Stain Buffer was added to wash the cells, followed by centrifugation at 500 × g for 5 minutes. Cells were resuspended in 400 μL of Stain Buffer and analyzed by flow cytometry.

### T cell isolation and activation

Mouse spleens were aseptically harvested and homogenized using a 70 μm cell strainer to generate a single-cell suspension. The cell suspension was transferred to a 50 mL centrifuge tube and centrifuged at 500 × g for 5 minutes. Five milliliters of erythrocyte lysis buffer (BD Biosciences) were added, and the mixture was incubated at room temperature for 5 minutes. Subsequently, 20 mL of PBS were added, followed by centrifugation at 500 × g for 5 minutes. The supernatant was discarded, and the spleen cells were resuspended in PBS. The cell suspension was filtered through a 40 μm cell strainer, counted, and centrifuged once more. The resulting cell pellet was resuspended in sorting buffer to a final concentration of 1 × 10⁸ cells/mL. Twenty microliters of Biotin-Antibody Mix were added, gently mixed, and incubated at 4°C for 10 minutes. After incubation, 200 μL of Streptavidin beads were added to the tube and incubated at 4°C for an additional 10 minutes. The tube containing the cell suspension was then placed on a magnetic stand for 5 minutes. The supernatant, containing the purified mouse CD8+ T cells, was collected for subsequent purification.

The isolated CD8+ T cells were plated at a density of 1 × 10⁶ cells/mL in 24-well tissue culture plates. Twenty-five microliters of pre-washed and resuspended Dynabeads® magnetic beads were added at a 1:1 bead-to-cell ratio. The cells were incubated at 37 °C in a humidified CO₂ incubator under the indicated experimental conditions. The tube was placed on a magnetic stand for 1-2 minutes to separate the beads from the solution. The supernatant containing the activated T cells was transferred to a new tube. The activated T cells were collected and used directly for further analysis.

### RNA-seq

SLC4A7 control and knockdown cells were washed, harvested on ice, lysed in TRIzol reagent, and transferred to nuclease-free microcentrifuge tubes for RNA extraction. RNA sequencing was performed by LC-Bio Technology (Hangzhou, China) for mRNA transcriptome analysis. After library construction, mRNA abundance was quantified using fragments per kilobase of transcript per million mapped reads (FPKM). Differential gene expression analysis was conducted using DESeq2 software. The Benjamini-Hochberg (BH) procedure was applied to control the false discovery rate, ensuring the reliability and reproducibility of the identified differentially expressed genes. Genes with a false discovery rate (FDR) < 0.05 and an absolute fold change > 1.5 were considered differentially expressed. Gene Ontology (GO) and Kyoto Encyclopedia of Genes and Genomes (KEGG) pathway enrichment analyses were subsequently performed. Bioinformatics analyses were performed using OmicStudio tools (https://www.omicstudio.cn/tool).

### Assay for Transposase Accessible chromatin using sequencing (ATAC-seq)

SLC4A7 control and knockdown cells were harvested at room temperature and counted using a Countstar Rigel S2 automated cell counter (Shanghai Ruiyu, China, FL20447). The required number of cells was lysed, and nuclei were isolated. Genomic DNA was purified by incubation in a transposition reaction containing Tn5 transposase. The resulting fragments were then amplified, with specific index sequences introduced during PCR. Magnetic bead-based size selection was performed to obtain library fragments of approximately 200-700 bp. Library concentration was quantified using a Qubit 3.0 fluorometer (Thermo Fisher Scientific), and fragment integrity was assessed using a Bioanalyzer 2100 (Agilent Technologies, USA). Sequencing was performed on an Illumina NovaSeq XP platform (Illumina, San Diego, CA, USA). Data analysis was conducted by LC-Bio Technology (Hangzhou, China).

### Luciferase assay

Blank cells were seeded into 24-well culture plates and incubated overnight to allow cell attachment and growth. Transfection was performed when cell confluence reached approximately 70%. CTCF control or overexpression plasmids were co-transfected into 293T cells together with PGL3-SLC4A7 promoter wild-type (WT) or mutant (MT) constructs and a Renilla luciferase plasmid. 48 hours post-transfection, cells were harvested, the culture medium was gently aspirated, and the cells were washed twice with PBS. Two hundred microliters of Cell Lysis Buffer were added to each well, followed by incubation on ice for 5-10 minutes. The lysates were then centrifuged at 15,000 × g for 5 minutes. Twenty microliters of the supernatant were transferred to each well of a white opaque 96-well plate. Subsequently, 100 μL of Dual-Lumi™ Firefly Luciferase Assay Reagent was added, and the plate was incubated at room temperature (approximately 25 °C) for 5 minutes. Chemiluminescence was measured using a multifunctional microplate reader. Next, 100 μL of Dual-Lumi™ Renilla Luciferase Assay Working Solution was added and mixed thoroughly. Chemiluminescence was subsequently measured using the same microplate reader. Relative light unit (RLU) values for both assays were compared, and the ratio of Firefly to Renilla luciferase activity was calculated to determine the degree of reporter gene activation.

### Chromatin immunoprecipitation (ChIP) assays

Chromatin immunoprecipitation (ChIP) assays were performed using the EZ-Magna ChIP® A/G Chromatin Immunoprecipitation Kit (Millipore, USA). All procedures were conducted according to the manufacturer's instructions. Briefly, on the day of the experiment, cells were treated with formaldehyde to crosslink protein to DNA. The cells were then lysed and sonicated to shear chromatin into DNA fragments ranging from 200 to 1000 bp. Immunoprecipitation was performed by incubating the lysate with a primary antibody against CTCF or a normal IgG control antibody overnight at 4°C. The following day, protein-DNA crosslinks were reversed. DNA was subsequently purified to remove chromatin-associated proteins, and prepared for downstream analysis. Enriched DNA fragments were detected by quantitative PCR (qPCR).

### Statistics and reproducibility

All statistical analyses were conducted using GraphPad Prism software (version X; GraphPad Software, San Diego, CA, USA). Statistical significance was assessed using two-tailed paired or unpaired Student's t-tests for comparisons between two groups, and one-way or two-way analysis of variance (ANOVA) for comparisons among more than two groups. Data are presented as mean±SD. Survival analyses were performed using the log-rank (Mantel-Cox) test. Specific sample sizes and statistical tests are detailed in the corresponding figure legends.

## Results

### Bicarbonate transporter SLC4A7 is highly expressed in NSCLC and is associated with poor clinical prognosis

**Figure 1:**
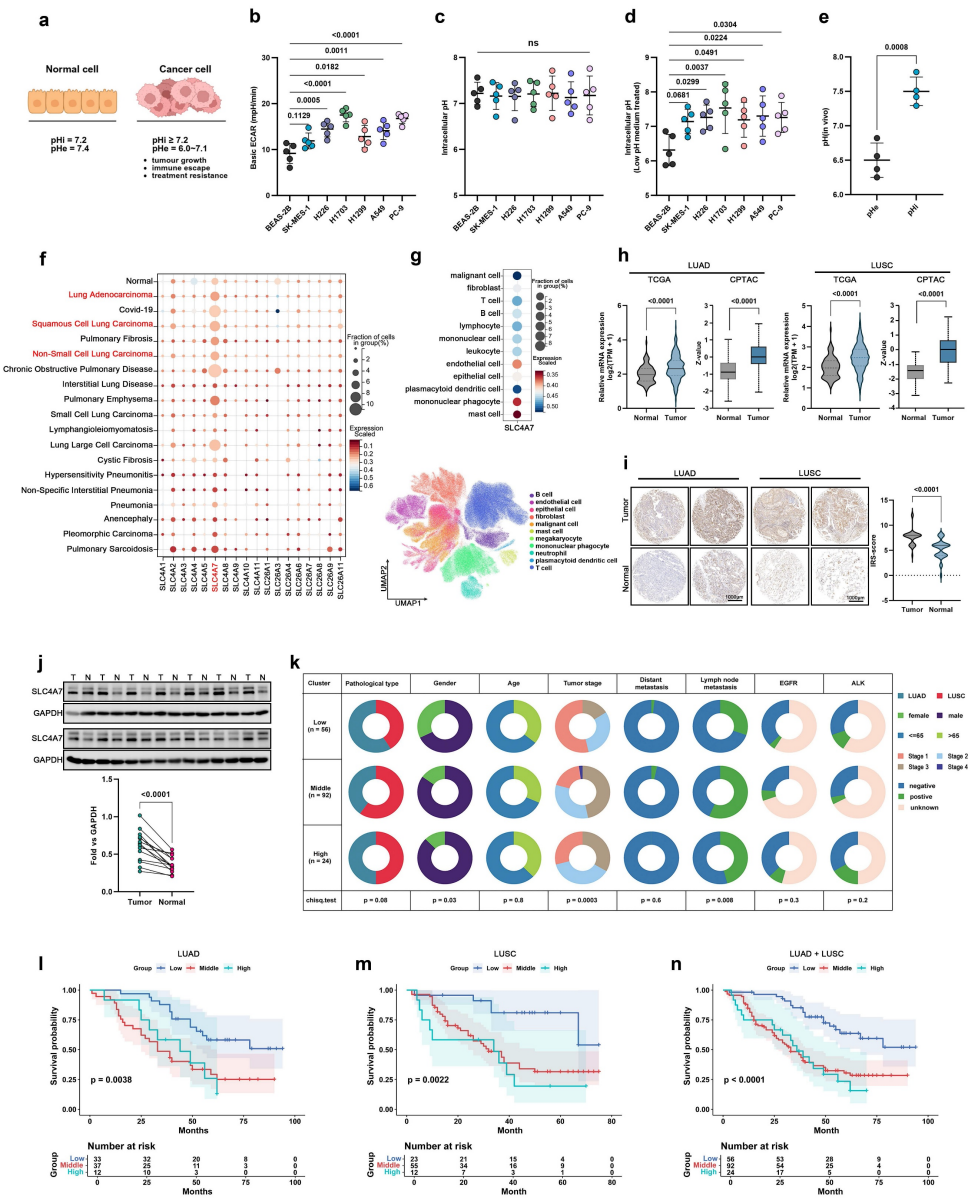
** SLC4A7 is the most expressed bicarbonate transporter in NSCLC and is associated with clinical prognosis. a,** Diagram of intracellular and extracellular pH patterns of normal cells and tumor cells. **b,** Seahorse experiment to detect extracellular basal acidification rate in lung normal and cancer cells (n = 5). **c,** Quantitative analysis of intracellular pH using pHrodo Red intracellular pH indicator dye (n = 5). **d,** Quantitative analysis of intracellular pH using pHrodo Red intracellular pH indicator dye with or without pH 6.5 medium treated (n = 5).** e,** Quantitative analysis of intracellular and extracellular pH values in subcutaneous tumor models using magnetic resonance spectroscopy (n = 4). **f,** Expression of different bicarbonate transporters in different lung diseases from publicly available single-cell databases (https://cellxgene.cziscience.com/). **g**, Expression of SLC4A7 in different cells from publicly available single-cell databases (https://cellxgene.cziscience.com/). **h,** Expression of SLC4A7 in tumor and adjacent tissues in TCGA and CPTAC databases. **i,** Expression of SLC4A7 in tumor and adjacent tissues in tissue microarray (n = 20), bar = 1000μm. **j,** Western blot analysis of SLC4A7 expression in tumor and normal tissue (n = 14). **k,** Clinical profile of tissue microarrays. **l-n,** Kaplan-Meier analysis on the prognostic relevance of SLC4A7 expression. *P* value was assessed by one-way ANOVA followed by the Tukey's post hoc **(b, c, d)** and two-tailed Student's t-test **(e, h-j)**.

Unlike normal cells, tumor cells exhibit a higher intracellular pH and a lower extracellular pH. Numerous studies have demonstrated that this specific acid-base environment is critical for supporting tumor growth, facilitating immune evasion, and contributing to treatment resistance **(Figure 1a)**. Extracellular acidification rate assays confirmed that non-small cell lung cancer (NSCLC) cell lines exhibited significantly higher extracellular acidification rates compared to epithelial cells **(Figure 1b)**. Furthermore, we utilized intracellular pH probes to evaluate the intracellular pH levels of epithelial and tumor cells after treatment with both normal (pH 7.4) and acidic (pH 6.5) culture media. The results demonstrated that the intracellular pH of tumor cells remained relatively alkaline **(Figure 1c, d)**, indicating that tumor cells have a greater capacity to withstand acidic environments. In addition, we established a subcutaneous tumor model and employed magnetic resonance spectroscopy (MRS) to assess the differences in intracellular and extracellular pH *in vivo*, further confirming that tumors possess a higher intracellular pH and lower extracellular pH **(Figure 1e)**.

The cellular transport of HCO₃⁻ is essential for maintaining intracellular and extracellular acid-base homeostasis and various physiological processes, a function that relies on multiple bicarbonate transporters^16^. Bicarbonate transporters are classified into two families: SLC4 and SLC26. The SLC4 family comprises ten genes, of which five SLC4A4, SLC4A5, SLA4A7, SLC4A8 and SLC4A10 are Na+-coupled bicarbonate transporters that mediate bicarbonate import across the plasma membrane^11^. Analysis of public single-cell datasets revealed that SLC4A7 is the most abundant bicarbonate transporter expressed in various lung diseases, including lung adenocarcinoma (LUAD) and squamous cell carcinoma (LUSC)** (Figure 1f)**. Notably, its expression was highest in malignant cells **(Figure 1g)**. Additionally, further analysis of the TCGA and CPTAC databases showed that only SLC4A7 was expressed and significantly upregulated in both LUAD and LUSC **(Figure 1h, Figure S1a-c)**. Immunohistochemical staining and western blot analysis of lung tissue samples further confirmed that SLC4A7 expression is markedly elevated in NSCLC **(Figure 1i, j)**. SLC4A7 expression was also upregulated in human NSCLC cell lines compared with normal lung epithelial cells **(Figure S1d)**.

The TCGA database also shows that SLC4A7 is elevated in cancers such as Cholangiocarcinoma, Esophageal carcinoma, Glioblastoma multiforme, Head and Neck squamous cell carcinoma, and Colon adenocarcinoma **(Figure S1e)**. Furthermore, construction of an immunohistochemical microarray comprising 172 cases revealed that elevated SLC4A7 expression was significantly associated with lymph node metastasis and poor survival outcomes **(Figure 1k-n, Figure S1f)**. Collectively, these data demonstrate that SLC4A7 serve as a major bicarbonate transporter in NSCLC and is strongly associated with clinical prognosis and patient survival.

### SLC4A7 knockdown alleviates TME acidification and inhibits tumor growth and metastasis

**Figure 2:**
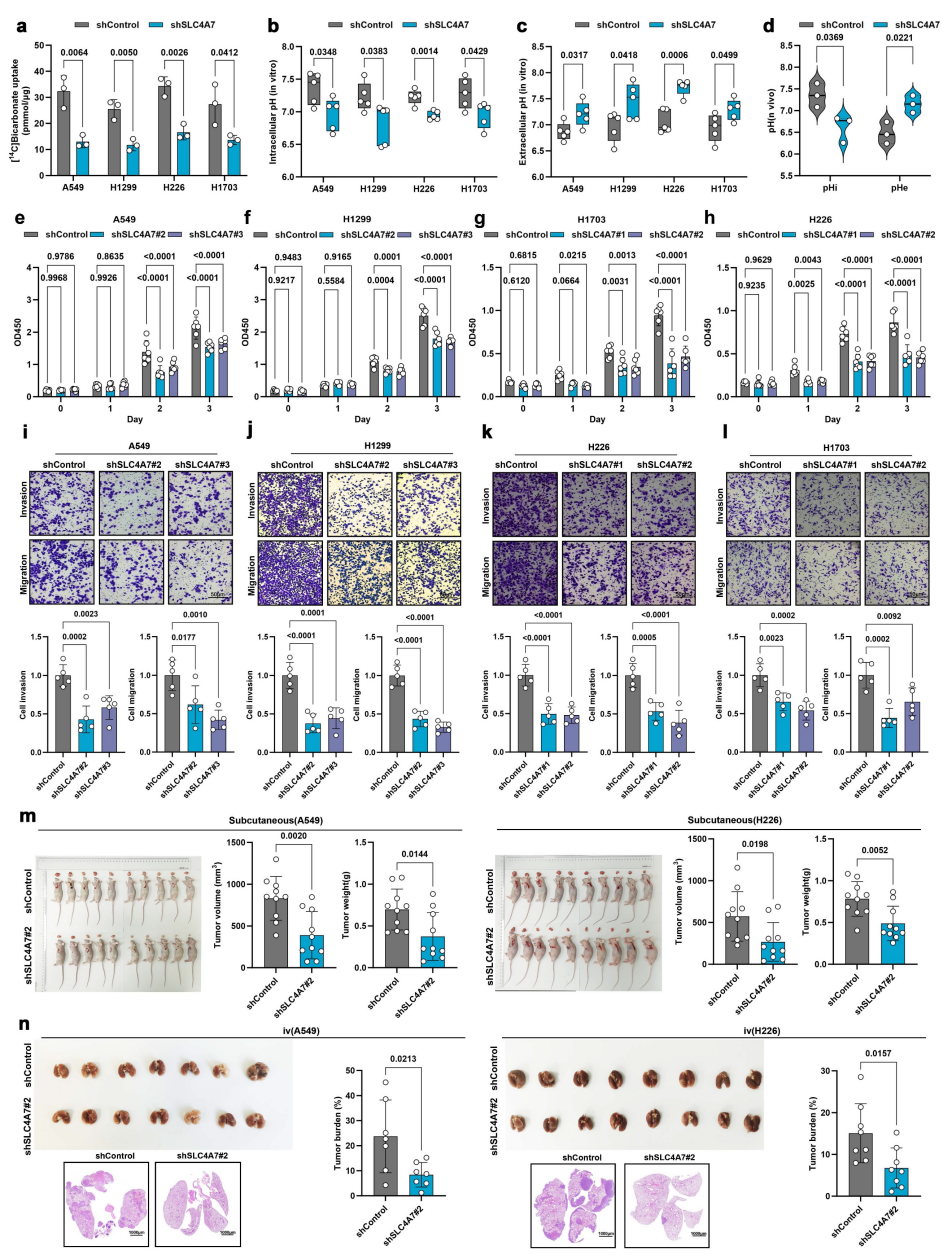
** SLC4A7 knockdown increases intracellular acidification and inhibits proliferation and metastasis of NSCLC. a,** [^14^C] bicarbonate uptake in shControl and shSLC4A7 cancer cells (n = 5). **b,** pHrodo Red intracellular pH indicator dye detects intracellular pH in tumor cells with or without SLC4A7 knockdown (n = 5). **c,** pH meter detects intracellular pH in tumor cells with or without SLC4A7 knockdown (n = 5). **d,** Quantitative analysis of intracellular and extracellular pH values in subcutaneous tumor models with or without SLC4A7 knockdown using magnetic resonance spectroscopy (n = 3). **e-h,** CCK8 assay was used to detect the proliferation ability of A549, H1299, H226 and H1703 cells after SLC4A7 knockdown. (n = 6). **i-l,** Representative images and statistical charts of traswell assay of A549, H1299, H226 and H1703 cells after SLC4A7 overexpression (n=5), bar = 50μm. **m,** Volume and weight of shControl and shSLC4A7 subcutaneous A549 and H226 tumors in nude mice (n = 10). **n,** Lung tumor burden after tail vein injection of shControl and shSLC4A7 A549 (right, n = 7) and H226 (left, n = 8) cells in nude mice. *P* value was assessed by two-tailed Student's t-test **(a-d, m, n)** and one-way ANOVA followed by the Tukey's post hoc **(e-l)**.

To further elucidate the role of SLC4A7 in the biological progression of NSCLC, we established stable SLC4A7 knockdown and overexpression cell lines in A549, H1299, H226, and H1703 cells **(Figure S2)**. Isotope-labeled uptake assays demonstrated that SLC4A7 knockdown significantly decreased bicarbonate uptake in all four NSCLC cell lines compared to controls **(Figure 2a)**. Furthermore, the reduction in bicarbonate uptake led to a decrease in intracellular pH and an increase in extracellular pH **(Figure 2b, c)**. Consistent results were observed in *in vivo* models **(Figure 2d)**.

We subsequently investigated the functional consequences of SLC4A7 knockdown on lung cancer progression. The proliferation of A549, H1299, H226, and H1703 cells was evaluated using CCK8 **(Figure 2e-h)**, EdU incorporation **(Figure S3a-h)**, and colony formation assays **(Figure S3i-l)**. The results indicated that SLC4A7 knockdown significantly inhibited the proliferation of NSCLC cell. Flow cytometry also showed that SLC4A7 knockdown increased the apoptosis rate of NSCLC cells **(Figure S3m-p)**. Transwell assays demonstrated that SLC4A7 knockdown markedly reduced the invasive and migratory capabilities of these cell lines **(Figure 2i-l)**. Epithelial-mesenchymal transition (EMT) endows tumor cells with migratory and invasive properties, representing a critical step in the initiation of metastasis. Western blot analysis demonstrated that the knockdown of SLC4A7 significantly ingcreased the levels of the epithelial marker E-cadherin while simultaneously reducing the expression of the mesenchymal markers N-cadherin and vimentin **(Figure S3q-t)**. *In vivo* experiments further confirmed that SLC4A7 knockdown suppressed NSCLC tumor growth and metastasis **(Figure 2m, n)**. Conversely, SLC4A7 overexpression promoted bicarbonate uptake as well as NSCLC cell proliferation and metastasis **(Figure S4)**. Collectively, these findings suggest that targeting SLC4A7 may alleviate tumor microenvironment acidification and suppress NSCLC growth and metastasis.

### RNA-seq and ATAC-seq analysis of SLC4A7 knockdown A549 cells

**Figure 3:**
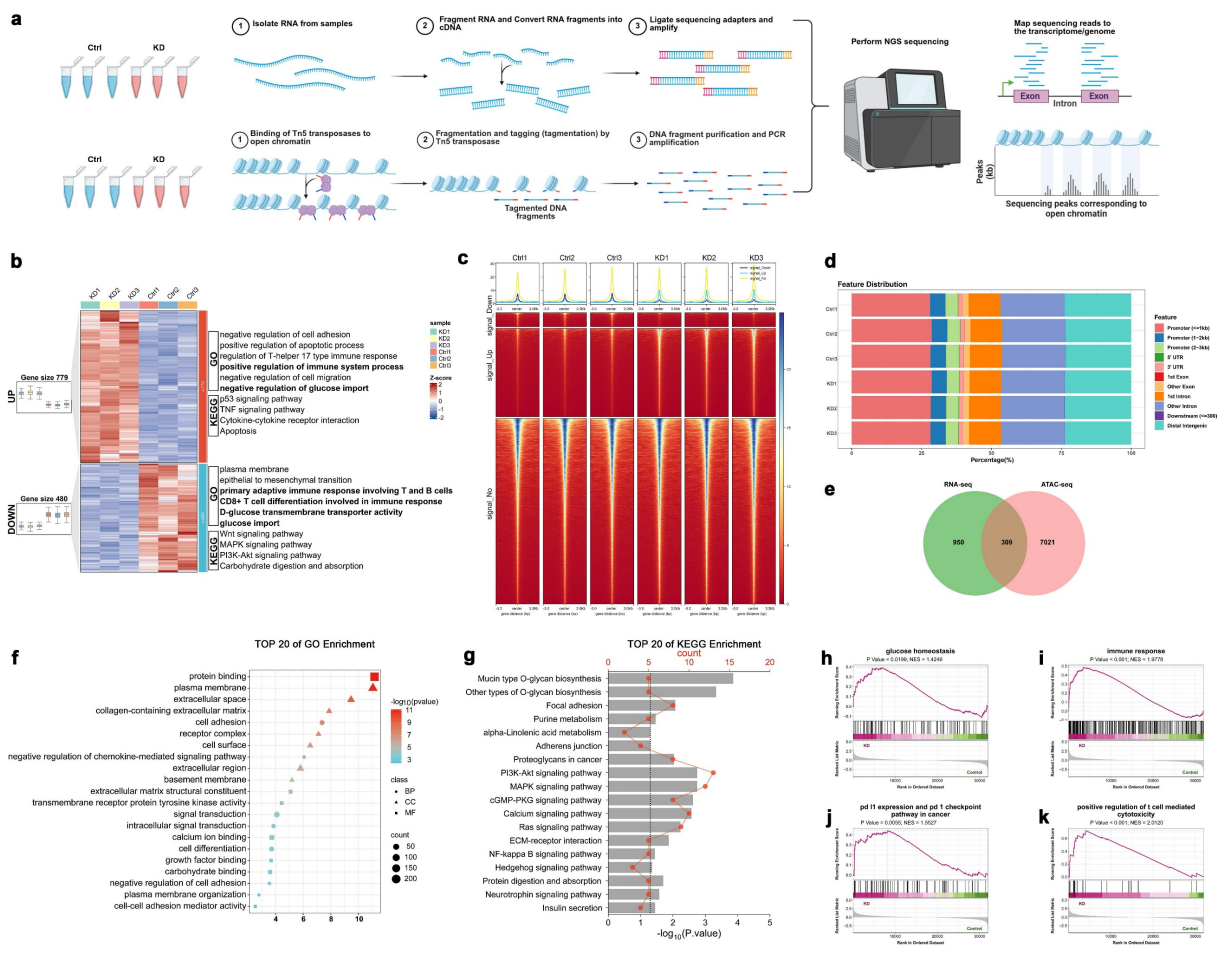
** Multi-omics analysis of SLC4A7 knockdown A549 cells by RNA-seq and ATAC-seq. a,** Schematic diagram of the multi-omics workflow of RNA-seq and ATAC-seq. **b,** Heatmap and functional annotation of differentially expressed genes in RNA-seq. **c,** Heatmap centered at ATAC-seq nucleosome-free peak summits for differential accessibility regions (DARs). **d,** Genomic distribution of DARs of A549 cells in the shControl and shSLC4A7 group. **e,** Venn diagram showing overlapping genes identified in RNA-seq and ATAC-seq of shSLC4A7 versus shControl A549 cells. **f,g,** Gene ontology (GO) enrichment and Kyoto Encyclopedia of Genes and Genomes (KEGG) analysis of significantly changed genes in shSLC4A7 versus shControl A549 cells. **h-k,** Gene set enrichment analysis of significantly differentially expressed mRNA with normalized enrichment score (NES) and *P* value.

To further elucidate the molecular mechanisms underlying the regulation of SLC4A7 in NSCLC biological functions, we performed transcriptomic analysis and an Assay for Transposase-Accessible Chromatin using sequencing (ATAC-seq) in SLC4A7 knockdown A549 cells **(Figure 3a)**. The transcriptomic analysis identified 779 upregulated and 480 downregulated genes (fold change ≥ 1.5, FDR < 0.05) **(Figure 3b)**. Gene Ontology (GO) and Kyoto Encyclopedia of Genes and Genomes (KEGG) pathway enrichment analyses revealed that the upregulated genes were significantly enriched in pathways related to positive regulation of the immune system and negative regulation of glucose transport, whereas the downregulated genes were predominantly associated with the plasma membrane, primary adaptive immune response involving T and B cells, CD8-positive T cell differentiation involved in immune response and glucose transport **(Figure 3b)**. ATAC-seq analysis identified 8,552 upregulated and 1,362 downregulated differentially accessible chromatin peaks (fold change ≥ 1.5, FDR < 0.05) **(Figure 3c, d)**. Integration of RNA-seq and ATAC-seq data identified 309 overlapping differentially expressed genes **(Figure 3e)**. GO and KEGG enrichment analyses of these genes indicated significant enrichment in pathways related to the plasma membrane, chemokine-mediated signaling, and carbohydrate binding **(Figure 3f, g)**. Gene set enrichment analysis (GSEA) further demonstrated that SLC4A7 knockdown significantly affected multiple biological processes in A549 cells, including glucose homeostasis, immune response, PD-L1 expression, the PD-1 checkpoint pathway in cancer, and positive regulation of T cell-mediated cytotoxicity **(Figure 3h-k)**. The results indicate that intracellular acidification and extracellular alkalinization resulting from SLC4A7 knockdown can directly influence tumor cell growth and metabolism, as well as impact the tumor immune microenvironment.

### SLC4A7 knockdown inhibits glycolysis in NSCLC cells

**Figure 4:**
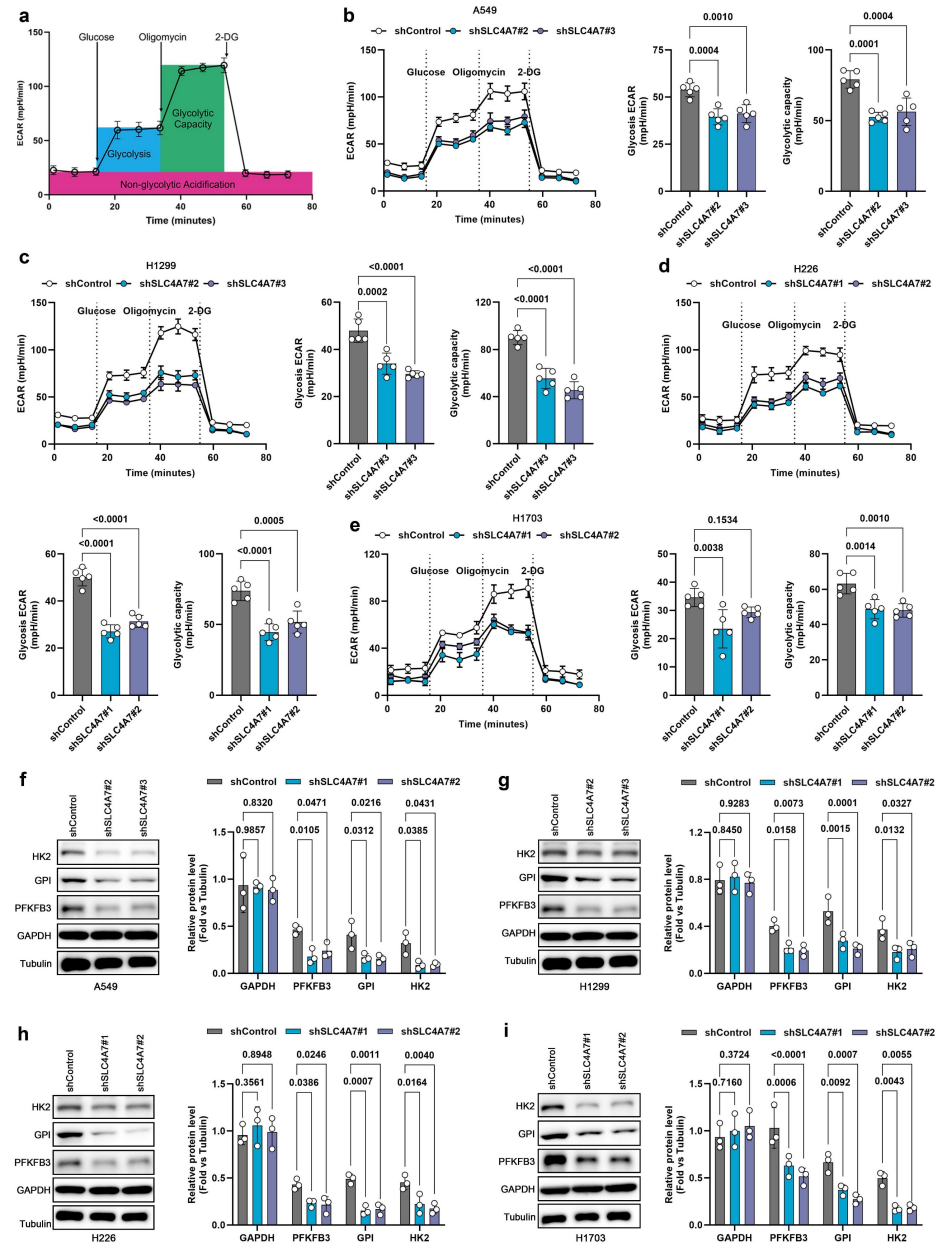
** SLC4A7 knockdown inhibits glycolysis in NSCLC cells. a,** Schematic diagram of the seahorse experiment. **b-e,** Seahorse experimental results showed that SLC4A7 knockdown reduced the level of glycolysis and the maximum glycolytic capacity of A549, H1299, H226 and H1703 cells (n = 5). **f-i,** Western blot analysis of the expression levels of key glycolytic proteins HK2, PFKFB3, GPI, and GAPDH in A549, H1299, H226, and H1703 cells with or without SLC4A7 knockdown (n = 3). *P* value was assessed by one-way ANOVA followed by the Tukey's post hoc **(b-e)** and two-way ANOVA with Tukey's multiple comparison test **(f-i)**.

Minor variations in pH can trigger significant metabolic alterations^37^, ^41^. RNA-seq and ATAC-seq analysis indicate that SLC4A7-mediated bicarbonate transport is involved in regulating glucose metabolism in lung cancer. To further validate the impact of SLC4A7 knockdown on glucose metabolism, we employed seahorse assays to assess glycolytic activity in A549, H1299, H226, and H1703 cells **(Figure 4a)**. The results demonstrated that SLC4A7 knockdown significantly decreased both the extracellular acidification rate (an indicator of glycolysis) and the maximal glycolytic capacity in all four cell lines **(Figure 4b-e)**. Furthermore, western blot analysis revealed that SLC4A7 knockdown markedly reduced the expression of key glycolytic enzymes, including HK2, GPI, and PFKFB3 **(Figure 4f-i)**. Conversely, SLC4A7 overexpression increased both glycolytic activity and the expression of HK2, GPI, and PFKFB3 in these cell lines **(Figure S5)**.

The observed reduction in glycolysis reflects the inhibition of glycolytic enzymes, such as hexokinase and 6-phosphofructokinase, due to decreased pHi. This inhibition establishes a negative feedback loop that protects cells from excessive intracellular acidification, given that glycolysis is a major source of proton generation^4^. Collectively, these data suggest that, in addition to directly impairing bicarbonate uptake, SLC4A7 inhibition induces metabolic changes that further decrease extracellular acidity by limiting proton production.

### Genetic and pharmacological inhibition of Slc4a7 promotes the infiltration of CD8+ T cells in NSCLC

**Figure 5:**
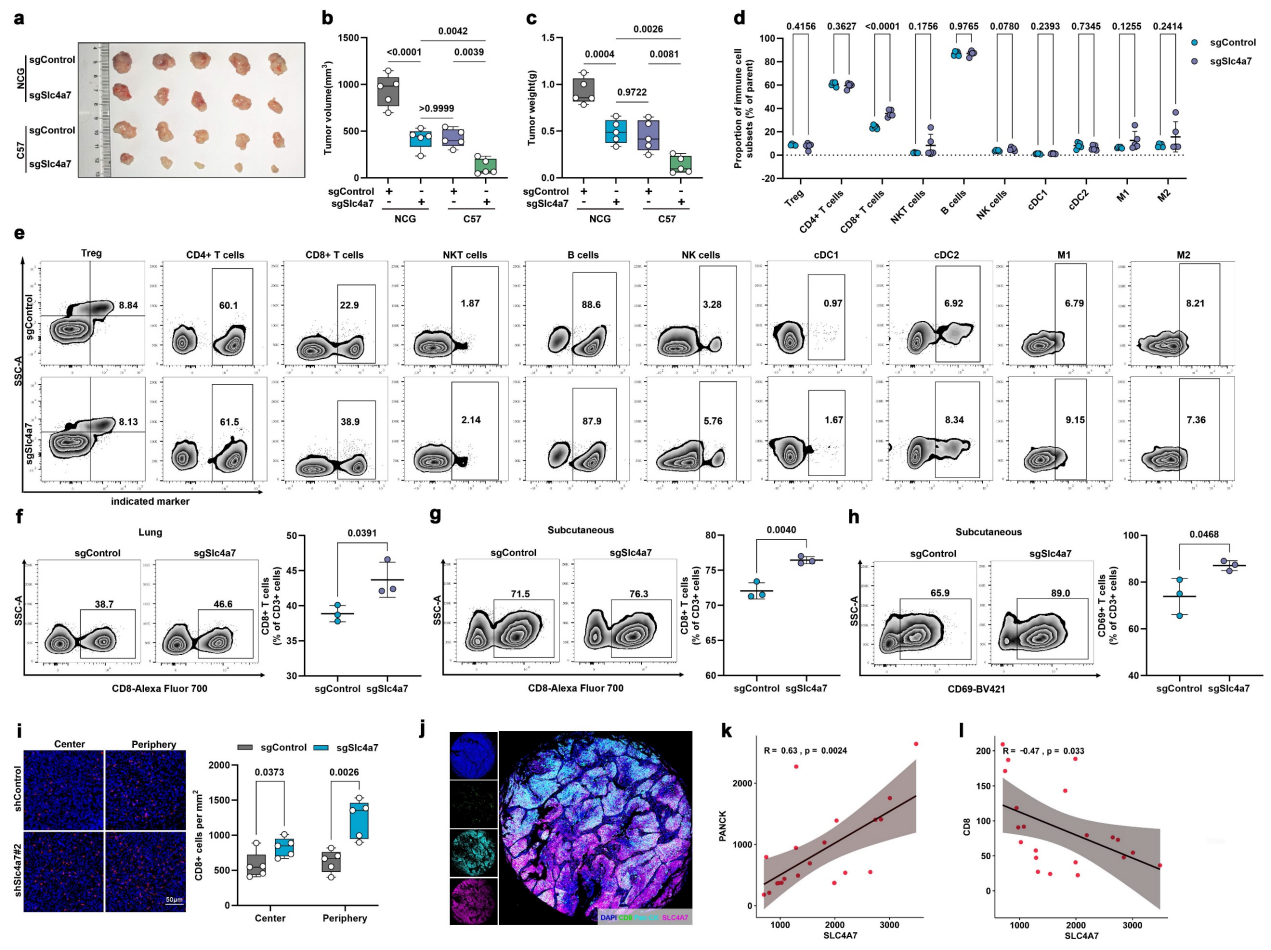
** Slc4a7 knockout enhances infiltration and activation of CD8+ T cells in tumors. a-c,** Volume and weight of sgControl and sgSlc4a7 subcutaneous KP tumors in Severe combined immunodeficiency mice (NCG mice) and C57 mice (n = 5). **d,e,** Flow cytometric analysis of the percentage of Treg, CD4 +, CD8 +, NKT, B, NK, cDC1, cDC2, M1 and M2 cells in sgControl and sgSlc4a7 spleen (n = 5). **f,g,** Flow cytometric analysis of the percentage of CD3+CD8+ T cells in sgControl and sgSlc4a7 orthotopic KP and subcutaneous KP tumors (n = 3). **h,** Flow cytometric analysis of the percentage of activated CD8+ T cells in sgControl and sgSlc4a7 subcutaneous KP tumors (n = 3). **i,** Representative images of infiltrating CD8+ T cells in KP subcutaneous tumors with or without Slc4a7 knockout (n = 5), bar = 50μm. **j,** Representative images of immunofluorescence staining in lung cancer tissues, bar = 1000μm. **k, l,** Immunofluorescence staining of SLC4A7 positive density in lung cancer tissues and its correlation with PANCK positive density, SLC4A7 positive density and CD8 positive density (n = 21). *P* value was assessed by unpaired, two-tailed Student's t-test.

Based on the established evidence for the association between tumor microenvironment pH and immune regulation and the results of our multi-omics analysis, we examined the subcutaneous tumor growth of KP cells with Slc4a7 knockout in both immunocompetent and severely immunodeficient mice. The results indicated that, in comparison to the immunodeficient group of mice, the sgSlc4a7 knockout group of mice with normal immunity exhibited a more significant reduction in both tumor volume and weight **(Figure 5a-c, S6a)**. To further identify the immune cells involved in this process, we employed flow cytometry to detect various immune cell types in the spleens of KP tumor-bearing mice, including regulatory T (Treg) cells, CD4+ T cells, CD8+ T cells, B cells, natural killer T (NKT) cells, natural killer (NK) cells, classical type 1 dendritic (cDC1) cells, classical type 2 dendritic (cDC2) cells, M1 macrophages, and M2 macrophages. The results indicated a significant increase in the proportion of CD8+ T cells within the Slc4a7 knockout group **(Figure 5d, e)**. We further used flow cytometry to detect the proportion of CD8+ T cells in in orthotopic lung cancer tissues and subcutaneous tumors, and obtained consistent results **(Figure 5f, g)**. Furthermore, the percentage of activated CD8+ T cells was significantly elevated in the Slc4a7 knockout subcutaneous tumor model **(Figure 5h)**. Immunofluorescence analysis further demonstrated increased infiltration of CD8+ T cells in both the tumor core and periphery **(Figure 5i)**. Additionally, Slc4a7 knockout also resulted in elevated serum and tumor concentrations of effector cytokines IFNγ and IL-2 **(Figure S6b)**.

In addition to genetic approaches, we used DIDS, a broad-spectrum SLC4A family inhibitor, to pharmacologically and systemically target Slc4a7. Consistent with Slc4a7 knockdown, SLC4A7 inhibitor DIDS treatment suppressed tumor growth *in vivo* and *in vitro* **(Figure S6c, d, e)** and increased intratumoral infiltration of CD8+ T cells as well as concentrations of IFNγ and IL-2 **(Figure S6f-h)**. Notably, CD8+ T cell depletion partially abrogated the differences in tumor growth observed in the subcutaneous KP model, further supporting a crucial role for CD8+ T cells following Slc4a7 targeting **(Figure S6i-k)**.

Subsequently, we analyzed the relationship between SLC4A7 expression and Pan-CK and CD8 in clinical samples using multicolor immunofluorescence. The results demonstrated that SLC4A7 expression was positively correlated with Pan-CK and negatively correlated with CD8 expression in clinical specimens **(Figure 5j-l)**.

### Targeting Slc4a7 improves T cell activation and function by reducing extracellular acidification

**Figure 6:**
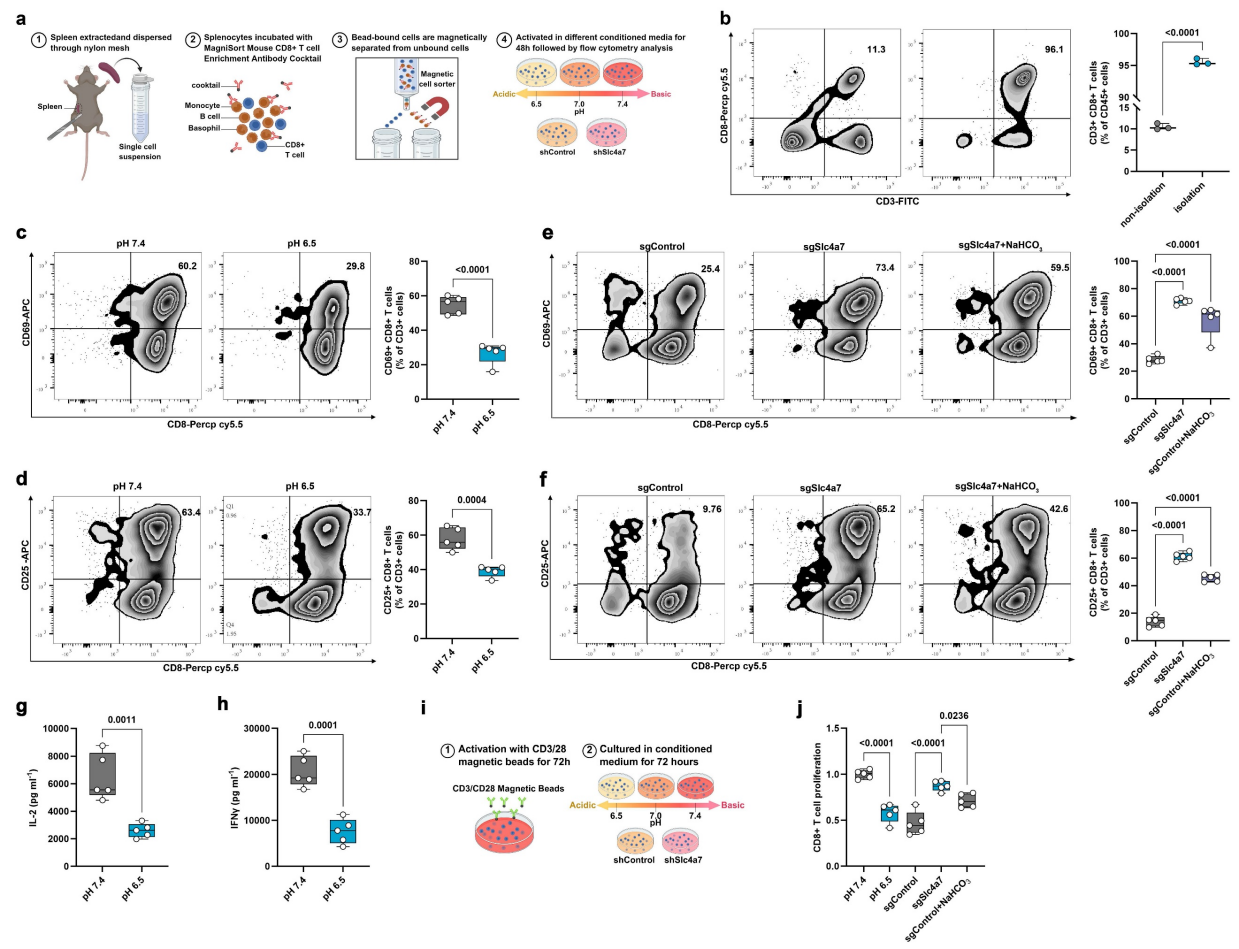
** Slc4a7 knockout-induced extracellular alkalization promotes the immune function of CD8+ T cells. a,** Schematic diagram of CD8+ extraction and activation experiment. **b,** Flow cytometry analysis of the purity of extracted CD8+ T cells (n = 3). **c,d,** Flow cytometry analysis of the proportion of CD69 and CD25 positive CD8+ T cells after 48 hours of culture under pH 7.4 and 6.5 conditions (n = 5). **e,f,** Flow cytometry analysis of the proportion of CD69 and CD25 positive CD8+ T cells after 48 hours of culture in shControl and shSlc4a7 conditioned medium (n = 5). **g,h,** ELISA analysis of IFNγ and IL2 secretion levels after 48 h of culture at pH 7.4 and 6.5 (n = 5). **i,j**, The proliferation of T cells in different conditioned media was analyzed by cell counting (compared with pH 7.4 group) (n = 5). *P* value was assessed by two-tailed Student's t-test **(b-d, g, h)** and one-way ANOVA followed by the Tukey's post hoc **(e, f, j)**.

To investigate the impact of Slc4a7-mediated alterations in extracellular pH on T cell activation *in vitro*, we isolated CD8+ T cells from mice using magnetic bead sorting **(Figure 6a, b)**. The isolated T cells were subsequently stimulated with CD3/CD28 magnetic beads for 48 hours under either acidic (pH 6.5) or physiological (pH 7.4) conditions. The proportion of CD25+ and CD69+ CD8+ T cells was markedly decreased in the acidic environment **(Figure 6c, d)**. Consistently, conditioned medium from control cells, compared to that from Slc4a7 knockout cells, significantly suppressed the expression of CD25 and CD69, both markers of T cell activation **(Figure 6e, f)**. Concurrently, the secretion of IFNγ and IL-2 was significantly impaired under acidic conditions (pH 6.5) **(Figure 6g, h)**. Supplementation with bicarbonate in the control group partially restored T cell activation **(Figure 6e, f)**. Furthermore, T cell proliferation was significantly inhibited in the acidic milieu **(Figure 6i, j)**.

It is well-established that activated T cells primarily rely on glycolysis for energy production. The enhanced glucose metabolism observed in tumor cells can lead to glucose depletion, thereby compromising the effector function of anti-tumor T cells^42^, ^43^. Therefore, we measured glucose concentrations in the culture medium of control and Slc4a7 knockdown groups and found significantly lower glucose levels in the control group **(Figure S7a)**. Moreover, glucose uptake by T cells in co-culture was significantly higher in the Slc4a7 knockout group compared to the control group **(Figure S7b, c)**. Depleting glucose from the culture medium resulted in a marked reduction in effector secretion and the expression of CD69 and CD25 **(Figure S7d-g)**, indicating that glucose utilization, and thus competition for this glucose, was directly regulating T cell effector function. Similar phenomena were observed in KP cell-conditioned medium when glucose was added or removed **(Figure S7h, i)**. These findings are consistent with our data on pH and glycolysis, suggesting that Slc4a7-deficient cancer cells modulate the extracellular environment to enhance T cell proliferation and activation.

**Figure 7:**
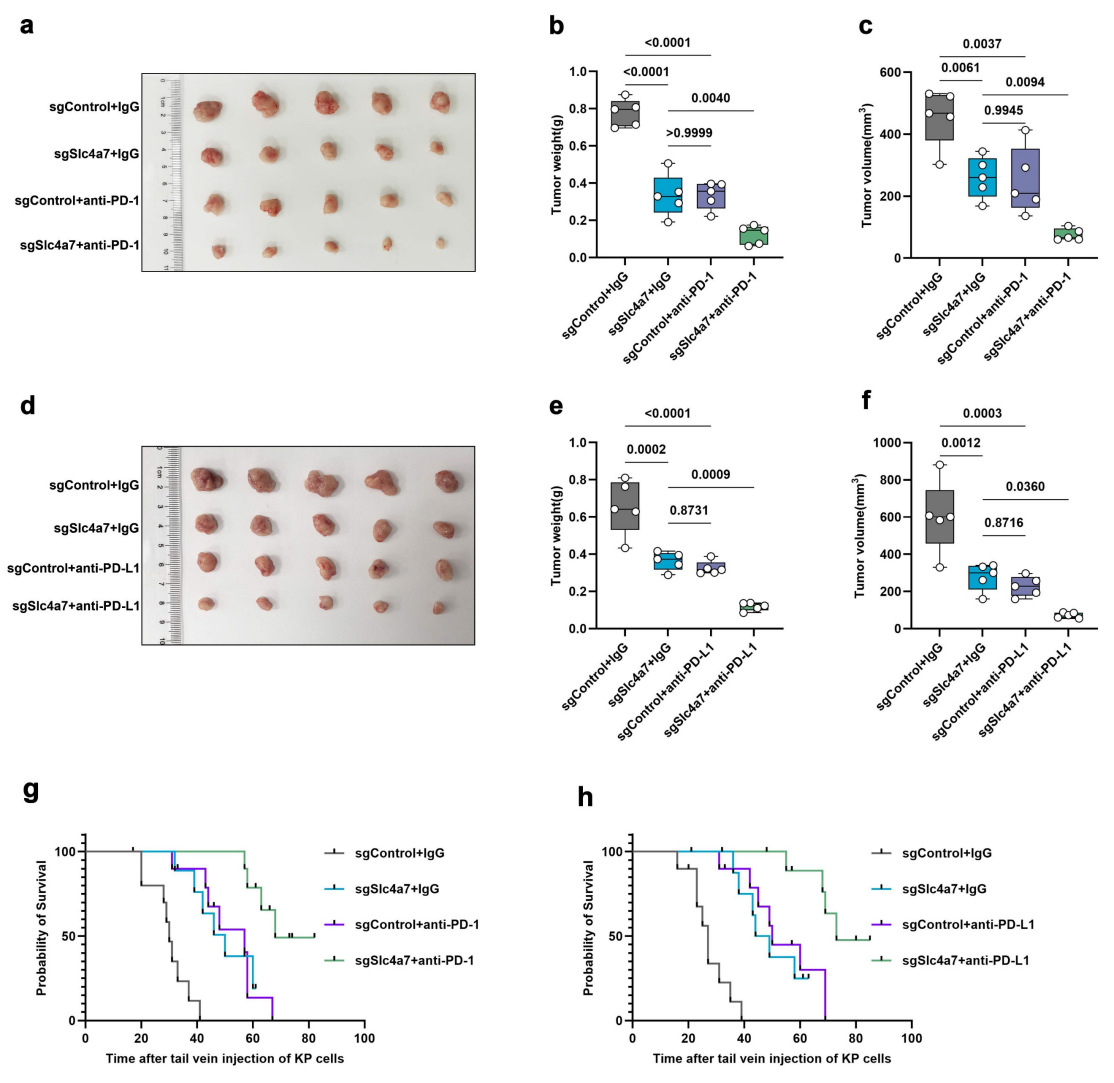
** Targeting Slc4a7 enhances PD1/L1 immunotherapy. a-f,** Weight and volume of sgControl and sgSlc4a7 subcutaneous KP tumors treated with anti-PD-1 and anti-PD-L1 (n = 5). **g,h,** Survival curves of mice bearing sgControl and sgSlc4a7 orthotopic KP tumors treated with anti-PD-1 and anti-PD-L1. *P* value was assessed by one-way ANOVA by the Tukey's post hoc (n = 10).

Given the role of Slc4a7 in immune regulation and the additional evidence from transcriptome GSEA suggesting that Slc4a7 influences immune responses, PD-L1 expression, the PD-1 checkpoint pathway in cancer, and positively regulates T cell-mediated cytotoxicity **(Figure 3h-k)**, we further explored the therapeutic potential of combining Slc4a7-targeted interventions with immunotherapy. To this end, we treated KP subcutaneous and orthotopic tumor models with anti-PD-1/PD-L1 immune checkpoint blockade (ICB). The results demonstrated that the combination of Slc4a7 inhibition and anti-PD-1/PD-L1 therapy produced a synergistic effect, resulting in significant tumor regression and prolonged survival **(Figure 7a-h)**. Collectively, these data suggest that the combination of Slc4a7 inhibition with ICB can significantly suppress tumor growth and extend survival.

### CTCF directly transcriptionally activates SLC4A7 expression

**Figure 8:**
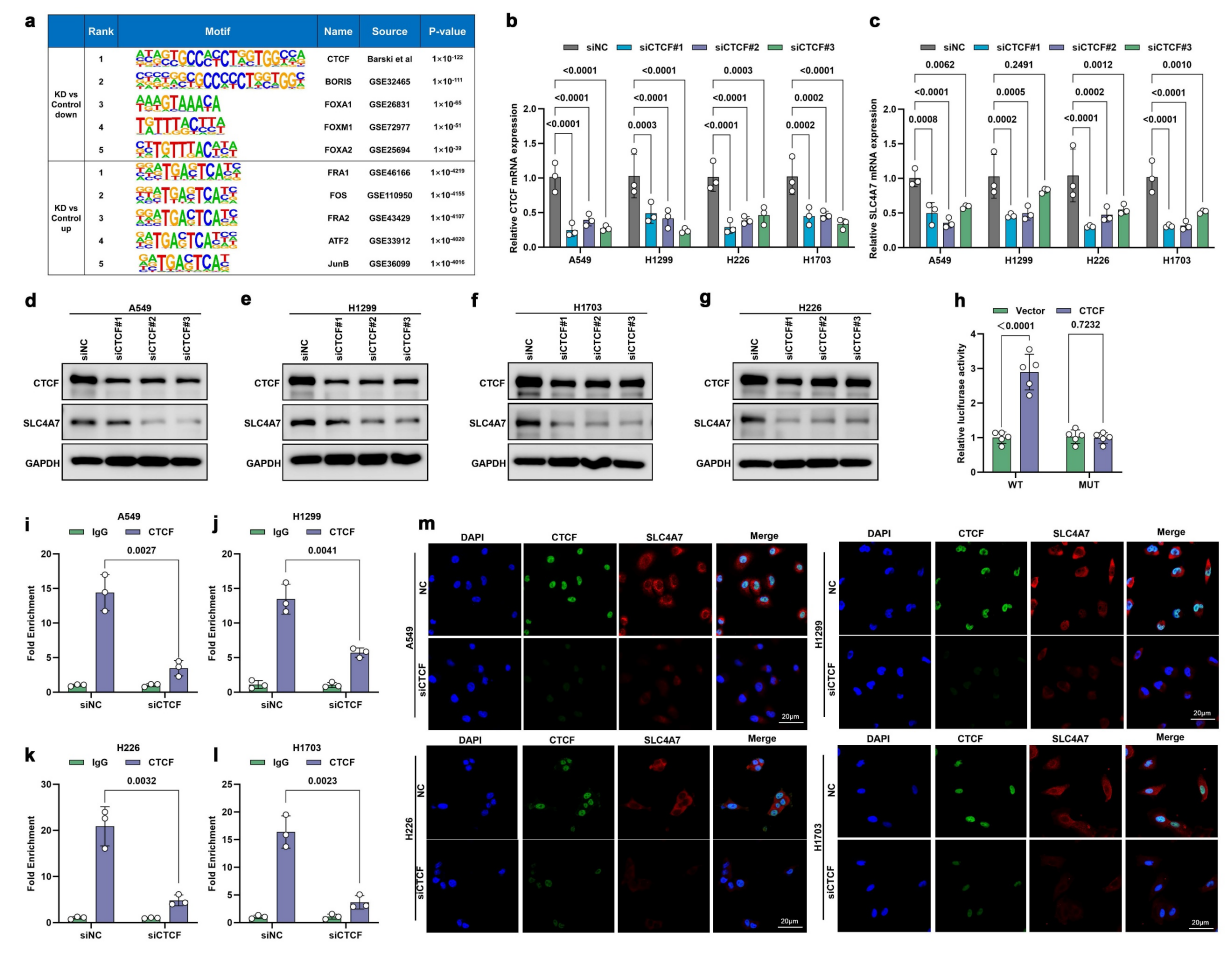
** CTCF directly activates the transcription of SLC4A7. a,** Homer Known Motif Enrichment analysis of the top transcription factor motifs enriched in DACRs between shControl and shSLC4A7 A549 cells. **b,c,** qPCR analysis of SLC4A7 expression with or without CTCF knockdown in A549, H1299, H226 and H1703 cells (n = 3). **d-g,** Western bot analysis of SLC4A7 expression with or without CTCF knockdown in A549, H1299, H226 and H1703 cells (n = 3). **h,** luciferase activity assay for detecting the binding ability of CTCF and SLC4A7. **i-l,** CHIP assay was used to analyze the binding ability of CTCF and SLC4A7 A549, H1299, H226 and H1703 cells with CTCF knockdown (n = 3). **m,** Representative immunofluorescence images of A549, H1299, H226 and H1703 cells with CTCF knockdown. bars = 20 μm. *P* value was assessed by two-way ANOVA with Tukey's multiple comparison test **(b, c)** and unpaired, two-tailed Student's t-test **(h-l)**.

We next investigated the underlying mechanisms regulating SLC4A7 expression. Further analysis of ATAC-seq data revealed that CTCF was the most significantly downregulated transcription factor **(Figure 8a)**. CTCF has been reported to be dysregulated in various cancers and may act as an oncogene in NSCLC development^44^, ^45^. Additional experiments demonstrated that CTCF knockdown reduced both SLC4A7 mRNA and protein expression **(Figure 8b-g)**. Moreover, analysis of TCGA data revealed that CTCF expression was upregulated in NSCLC tissues compared to adjacent normal tissues and positively correlated with SLC4A7 expression, suggesting that CTCF may serve as a key regulator of SLC4A7 **(Figure S8a-c)**. To identify the CTCF binding region within the SLC4A7 promoter, a pGL3 luciferase plasmid containing the SLC4A7 promoter was constructed and transfected into HEK-293T cells. Dual-luciferase reporter assays demonstrated that CTCF overexpression significantly increased luciferase activity at wild-type (WT) binding sites compared to controls **(Figure 8h)**. Chromatin immunoprecipitation (ChIP) assays further confirmed CTCF binding within the SLC4A7 promoter region **(Figure 8i-l)**. Immunofluorescence staining additionally corroborated these findings **(Figure 8m)**. Collectively, these data indicate that SLC4A7 expression is transcriptionally activated by CTCF.

In addition, we investigated the functional significance of CTCF in NSCLC. Western blot analysis showed that CTCF was highly expressed in lung cancer tissues and lung cancer cells **(Figure S8d, e)**. TCGA data analysis also showed that high CTCF expression is associated with poor prognosis **(Figure S8f, g)**. Functional experiments indicated that CTCF knockdown resulted in decreased intracellular pH and increased extracellular pH **(Figure S9a, b)**. Furthermore, knockdown of CTCF suppressed proliferation, invasion, and migration of A549, H1299, H226, and H1703 cells **(Figure S9c-j)**. Subsequent rescue experiments demonstrated that SLC4A7 overexpression antagonized the effects of CTCF knockdown on intracellular and extracellular pH **(Figure S9a, b)**, and reversed the inhibition of NSCLC cell proliferation, invasion, and migration **(Figure S9c-j)**. Taken together, these results suggest that CTCF promotes the progression of NSCLC primarily through the regulation of SLC4A7 expression.

## Discussion

In solid tumors, bicarbonate transport undergoes significant reprogramming to maintain intracellular pH homeostasis and support tumor proliferation, metabolism, and immune evasion^2^, ^4^. Our data demonstrate that SLC4A7 is the most critical bicarbonate transporter in NSCLC. It is highly expressed in NSCLC and mediates the import of HCO₃⁻ into the cell, thereby maintaining an acid-base microenvironment conducive to tumor progression. Inhibition of SLC4A7 not only results in intracellular acidification and a reduction in glycolysis, but it also induces extracellular alkalinization, which promotes CD8+ T cell infiltration and cytotoxicity and enhances the efficacy of immune checkpoint inhibitors.

Bicarbonate transporters are essential for maintaining acid-base homeostasis in normal tissue function throughout the body^11^, ^16^. Recent studies have demonstrated that these transporters play a crucial role in tumor progression. Yang *et al.* reported that elevated expression of SLC4A4 is closely associated with poor prognosis in breast and prostate cancers^46^, ^47^. Additionally, Cappellesso *et al.* demonstrated that inhibiting SLC4A4 suppresses pancreatic cancer progression by reducing extracellular acidification through increased bicarbonate accumulation^38^. Furthermore, SLC4A7 is upregulated in breast, pancreatic, and head and neck squamous cell carcinomas, with its elevated expression correlating with poor prognosis^17^, ^19^, ^48^. However, limited research has been conducted on bicarbonate transporters in NSCLC. In our study, we found that SLC4A7 is upregulated in NSCLC and is associated with poor prognosis. Our *in vitro* and *in vivo* data also indicated that inhibiting SLC4A7 significantly suppresses NSCLC proliferation and metastasis. These findings emphasized the critical role of SLC4A7 in the progression of NSCLC.

The interaction between tumor glycolysis and microenvironmental pH is bidirectional, as low intracellular pH inhibits enzymes involved in glycolysis, which may consequently limit the malignant behavior of cancer cells^2^, ^8^, ^9^. In this study, our *in vitro* and *in vivo* data have demonstrated that SLC4A7 contributes to the regulation of intracellular and extracellular pH during NSCLC progression. Inhibition of SLC4A7 impairs bicarbonate uptake by tumor cells, resulting in its accumulation in the extracellular space and a consequent increase in pHe. Additionally, SLC4A7 knockdown in tumor cells compromises intracellular buffering capacity, leading to decreased intracellular pH (pHi) and subsequently a reduction in glycolytic activity. Diminished glycolysis lowers the rate of extracellular acidification, further ameliorating extracellular pH by reducing proton export. Conversely, cancer cells with intact SLC4A7 expression maintain optimal pHi through sustained bicarbonate import, thereby preventing acidosis-induced inhibition of glycolysis. This establishes a feed-forward loop wherein bicarbonate uptake from the extracellular space not only lowers pHe but also facilitates glycolysis by sustaining pHi, thereby promoting proton export and further acidifying the extracellular environment. Through this mechanism, NSCLC cells maintain an alkaline intracellular pHi and an acidic extracellular pHe.

An acidic tumor microenvironment not only affects cancer cells directly but also alters the composition and function of immune cells within the TME^4^, ^21^-^24^. CD8 + cytotoxic T cells represent the most potent anti-tumor immune subset, capable of recognizing and eliminating malignant cells. An acidic microenvironment can also induce functional impairment in CD8⁺ T cells and natural killer cells, and diminish the production of key effector cytokines such as IFNγ, IL-2, and TNFα^31^-^34^. Our full spectrum flow cytometry data demonstrated that knockout Slc4a7 expression increases both the abundance and activation of tumor-infiltrating CD8⁺ T cells, as well as the levels of effector cytokines IFNγ and IL-2. *In vitro* experiments further reveal that Slc4a7-mediated reduction in pHe inhibits CD8⁺ T cell activation and effector cytokine secretion. Furthermore, since activated T cells primarily depend on glycolysis, tumor cells suppress T cell activation by depleting glucose^29^, ^42^, ^43^. Consistent with these findings, our data show that Slc4a7 inhibition limits both tumor cell glycolysis. Collectively, our studies indicate that targeting Slc4a7 enhances CD8⁺ T cell function by increasing pHe within the tumor microenvironment, and provides a metabolic advantage to CD8⁺ T cells through reduced tumor cell glucose consumption.

Lung cancer remains the leading cause of cancer-related mortality worldwide^49^. In recent decades, immunotherapy has revolutionized the treatment of lung cancer. Immune checkpoint inhibitors, particularly those targeting PD-1/PD-L1 and CTLA-4, have significantly improved patient survival outcomes^50^. However, two major challenges persist in clinical practice: some patients exhibit primary resistance (failure to respond to immune checkpoint inhibitors), while most initial responders eventually develop acquired resistance, resulting in disease relapse^51^, ^52^. Previous studies have demonstrated that neutralizing TME acidification can enhance immunotherapy responses across various tumor types, including melanoma, breast cancer and oesophageal adenocarcinoma^29^, ^30^, ^33^, ^53^. Our findings reveal that inhibition of SLC4A7 enhances the efficacy of PD-1/L1 immunotherapy in NSCLC, resulting in complete or partial regression of KP tumors and prolonged survival, thereby further underscoring the therapeutic potential of targeting SLC4A7.

Given the critical role of pH regulation in tumor progression, preventing tumor acidity has been evaluated as a strategy to limit tumor growth and metastasis. To date, strategies aimed at improving tumor pH have primarily focused on reducing extracellular acidity by inhibiting glycolysis, thereby suppressing the production of acidic metabolites^29^, ^35^, ^54^. However, these approach not only affect cancer cell metabolism but also influence the metabolic activity of antitumor immune cells within the TME. Robust glycolytic metabolism is crucial for the proliferation and activation of antitumor immune cells^55^; consequently, while previous strategies have successfully lowered TME acidity, they have inadvertently compromised antitumor immune responses. As cancer cells in acidic TMEs depend on net acid extrusion for survival, inhibiting these adaptive mechanisms, particularly bicarbonate transporters, represents a promising therapeutic avenue. In this study, targeting SLC4A7 specifically disrupts bicarbonate transport in cancer cells and enhancing the efficacy of immune checkpoint blockade. Furthermore, transcriptomic and ATAC-seq analyses have identified CTCF as an upstream transcriptional regulator of SLC4A7. Therefore, targeting bicarbonate transporters, along with molecules linking tumor acidosis to increased cancer invasiveness, represents a novel therapeutic strategy to inhibit tumor progression and metastasis. This study acknowledges certain limitations. Due to the unavailability of specific inhibitors, we exclusively employed pan-SLC4A family inhibitors. The lack of specific inhibitors has constrained both our research and the clinical application targeting this pathway. Therefore, our future study will focus on the development of specific inhibitors.

## Conclusions

In summary, our studies demonstrate that targeting SLC4A7 disrupts the specialized acid-base microenvironment of tumors and enhances the function and anti-tumor effects of tumor-infiltrating CD8⁺ T cells. These results pave the way for SLC4A7-based therapeutic strategies that have the potential to alleviate tumor acidosis, overcome immunosuppression, enhance CD8⁺ T cell fitness, and increase the sensitivity of NSCLC to current immunotherapy regimens.

## Supplementary Material

Supplementary figures.

## Data Availability

All data can be obtained by contacting the corresponding author.

## Abbreviations

TME: Tumor Microenvironment
SLC4A7: Solute Carrier Family 4 Member 7
TCGA: The Cancer Genome Atlas
CPTAC: Clinical Proteomic Tumor Analysis Consortium
LUAD: Lung Adenocarcinoma
LUSC: Squamous Cell Carcinoma
CCK-8: Cell Counting Kit-8
EMT: Epithelial-Mesenchymal Transition
pHe: Extracellular pH
pHi: Intracellular pH
Nbcn1: Na⁺/HCO₃⁻ Cotransporter 1
STR: Short Tandem Repeat
MRS: Magnetic Resonance Spectroscopy
ECM: Extracellular Matrix
GO: Gene Ontology
KEGG: Kyoto Encyclopedia of Genes and Genomes
GSEA: Gene Set Enrichment Analysis
ICB: Immune Checkpoint Blockade
Chip: Chromatin Immunoprecipitation
