# Supplementary material for: SLC4A7 Drives NSCLC Progression and Immune Evasion via pH Dysregulation: Its Targeting Synergizes with Anti-PD-1/L1 Therapy
Source: Int J Biol Sci. 2026 Mar 30;22(8):3950–68. doi: 10.7150/ijbs.129129 (PMC13137449; doi:10.7150/ijbs.129129)
Supplement: Supplementary file 1 — Supplementary figures. [file ijbsv22p3950s1.pdf]

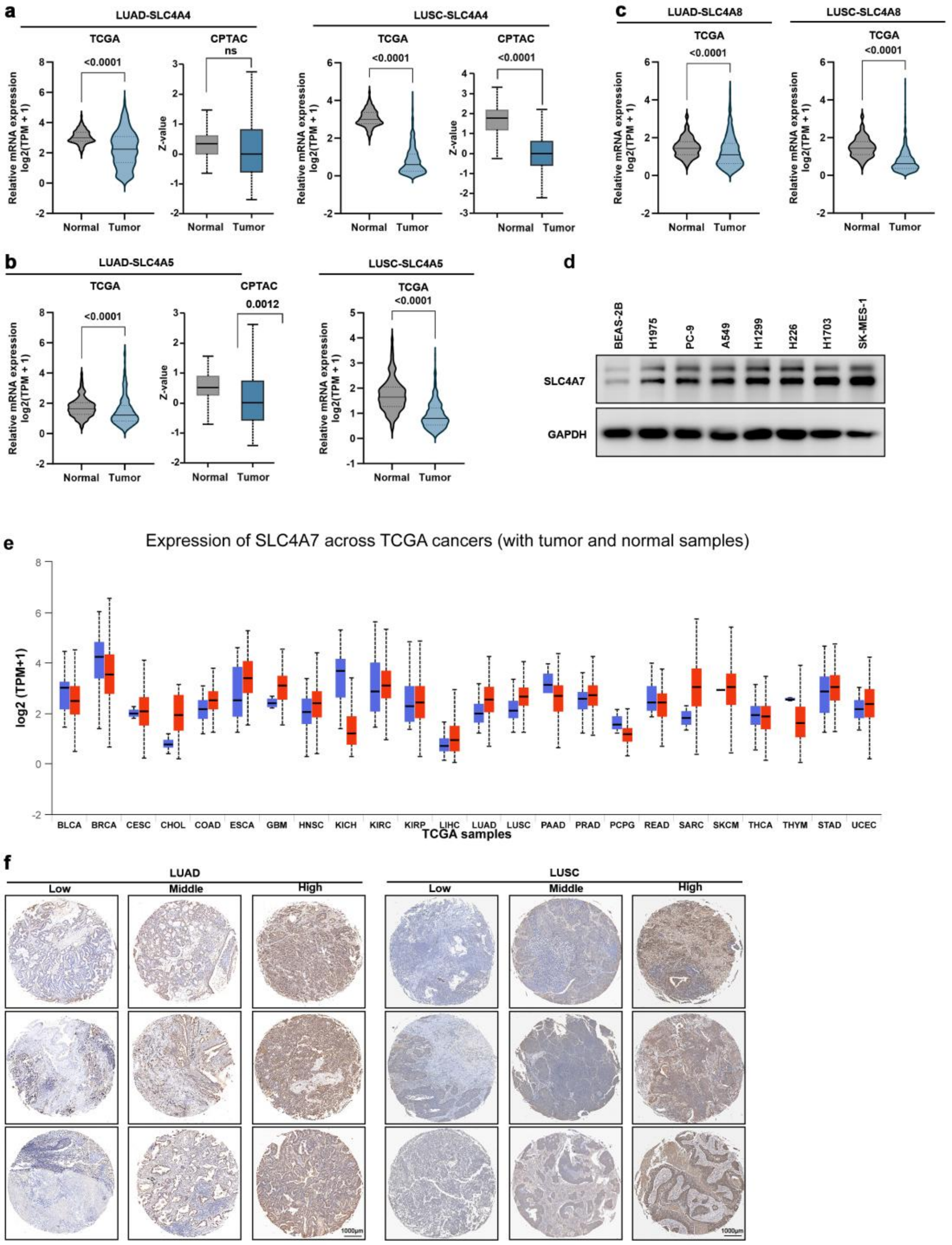

Figure S1. SLC4A7 expression is elevated in NSCLC

a-c, Expression of SLC4A4 (**a**), SLC4A5 (**b**) and SLC4A8 (**c**) in tumor and normal tissues in TCGA and CPTAC databases. **d,** Representative images of western blot analysis of SLC4A7 expression in BEAS-2B, H1975, PC-9, A549, H1299, H226, H1703 and SK-MES-1 cells **e,** Expression of SLC4A7 in various tumors in the TCGA database. **d,** Representative images of immunohistochemical staining of SLC4A7 in NSCLC, bar =

1000µm. *P* value was assessed two-tailed Student's t-test.

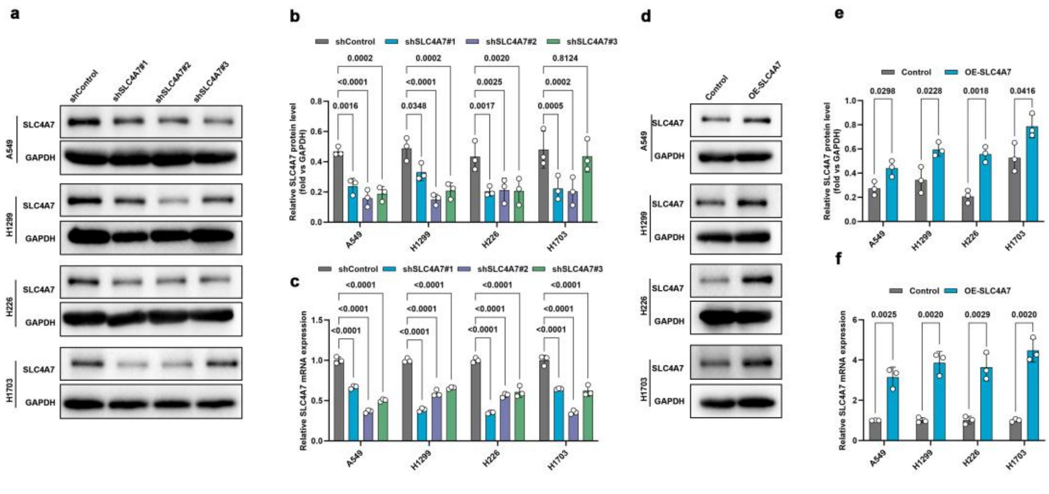

Figure S2. Construction of stable cell lines with overexpression and knockdown of SLC4A7

a-c, Western blot (**a, b**) and qPCR (**c**) analysis of SLC4A7 expression in A549, H1299, H226 and H1703 cells with or without SLC4A7 knockdown (n = 3). **d-f,** Western blot (**d, e**) and qPCR (**c**) analysis of SLC4A7 expression in A549, H1299, H226 and H1703 cells with or without SLC4A7 overexpression (n=3). *P* value was assessed by two-way ANOVA with Tukey's multiple comparison test **(a-c)** and two-tailed Student's t-test **(d-f)**.

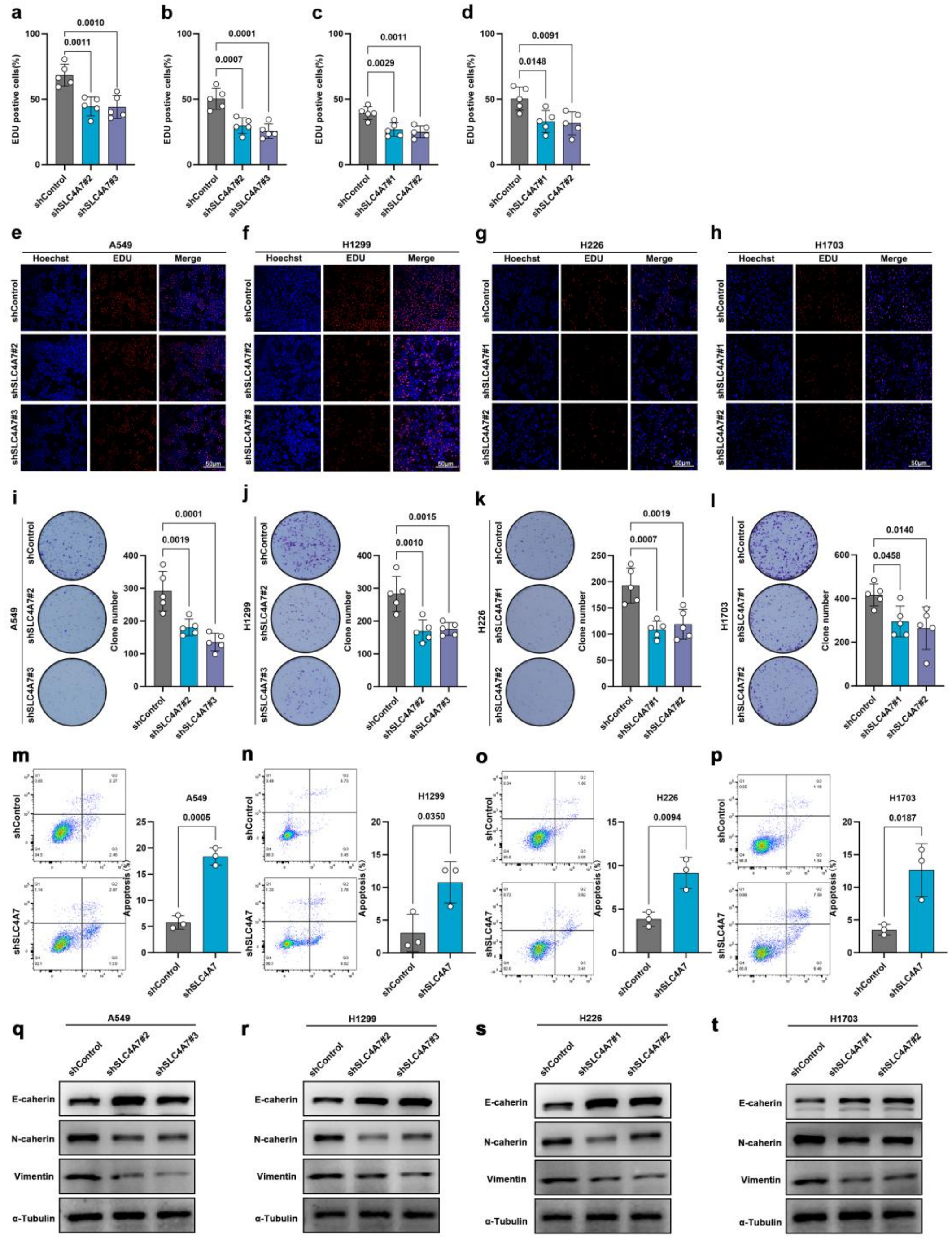

Figure S3. SLC4A7 knockdown inhibits proliferation of non-small cell lung cancer cells

a-d, EDU assay for detecting the proliferation of A549, H1299, H226 and H1703 cell with or without SLC4A7 knockdown (n = 5). **e-h,** Representative images of EdU staining of A549, H1299, H226 and H1703 cells after SLC4A7 knockdown (n=5), bar = 50μm. **i-l,** Representative images and statistical charts of clone formation of A549, H1299, H226 and H1703 cells after SLC4A7 knockdown (n=5). **m-p,** Flow cytometry for detecting the

apoptosis rate of A549, H1299, H226 and H1703 cell with or without SLC4A7 knockdown (n = 3). **q-t,** Representative images of Western blot analysis of E-cadherin, N-cadherin, and vimentin expression in A549, H1299, H226, and H1703 cells with or without SLC4A7 knockdown. *P* value was assessed by one-way ANOVA followed by the Tukey's post hoc.

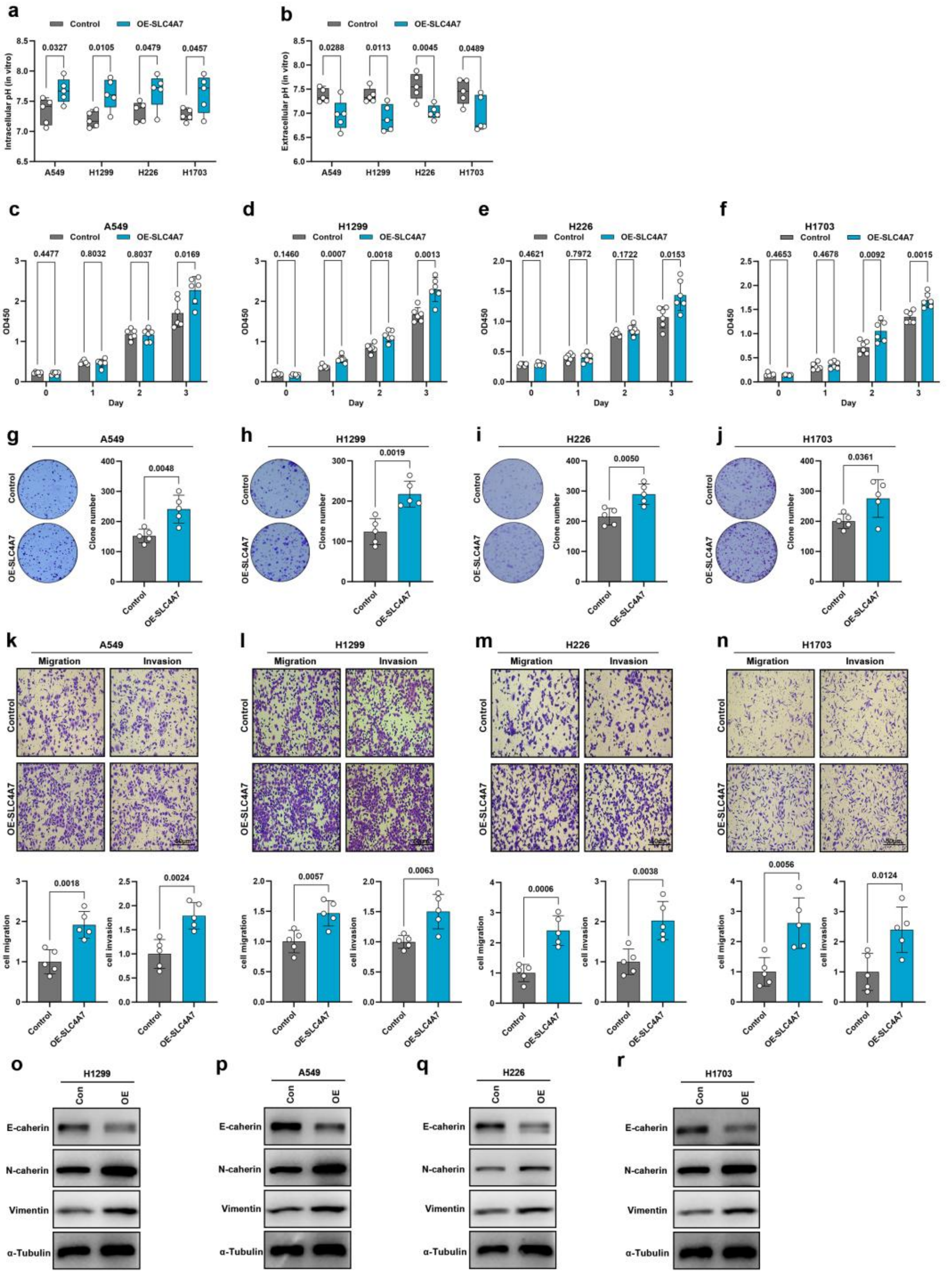

Figure S4. SLC4A7 overexpression promotes proliferation and metastasis of NSCLC cells

a, Quantitative analysis of intracellular pH using pHrodo Red intracellular pH indicator dye in A549, H1299, H226 and H1703 cells with or without SLC4A7 overexpression (n=5). **b,** Quantitative analysis of extracellular pH values using single-barreled H+-sensitive microelectrodes in A549, H1299, H226 and H1703 cells with or without

SLC4A7 overexpression (n=5). **c-f,** CCK8 assay was used to detect the proliferation ability of A549, H1299, H226 and H1703 cells after SLC4A7 overexpression (n = 6). **g-j,** Representative images and statistical charts of clone formation of A549, H1299, H226 and H1703 cells after SLC4A7 overexpression (n=5). **k-n,** Representative images and statistical charts of traswell assay of A549, H1299, H226 and H1703 cells after SLC4A7 overexpression (n=5), bar = 50μm. **o-r,** Western blot analysis of E-cadherin, N-cadherin and Vimentin expression levels with or without SLC4A7 overexpression (n = 3). *P* value was assessed by two-tailed Student's t-test.

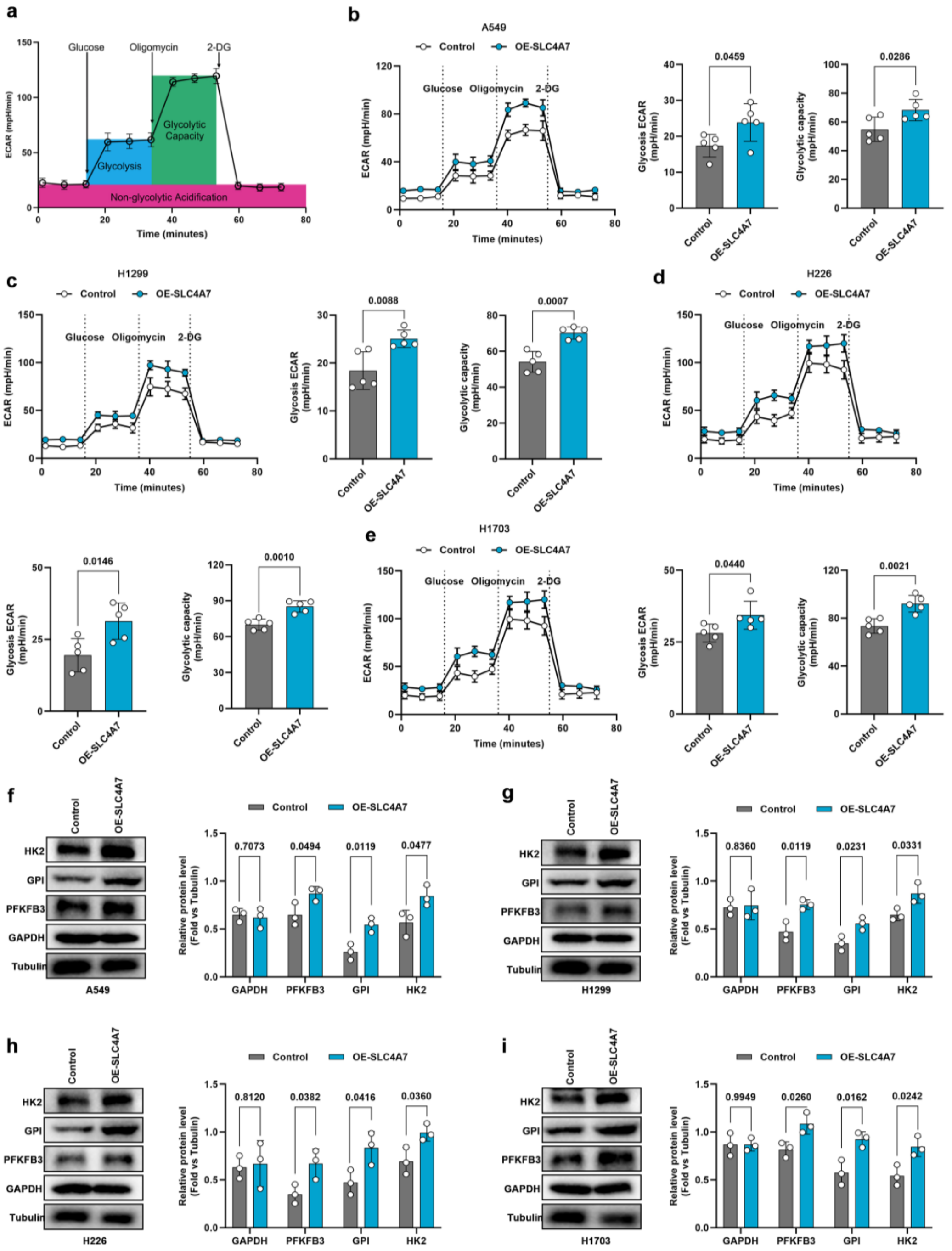

Figure S5. SLC4A7 overexpression promotes glycolysis in NSCLC cells

a, Schematic diagram of the seahorse experiment. **b-e,** Seahorse experimental results showed that SLC4A7 overexpression increased the level of glycolysis and the maximum glycolytic capacity of A549, H1299, H226 and H1703 cells (n = 5). **f-i,** Western blot analysis of the expression levels of key glycolytic proteins HK2, PFKFB3, GPI, and GAPDH in A549, H1299, H226, and H1703 cells with or without SLC4A7 overexpression (n = 3). *P* value was assessed two-tailed Student's t-test.

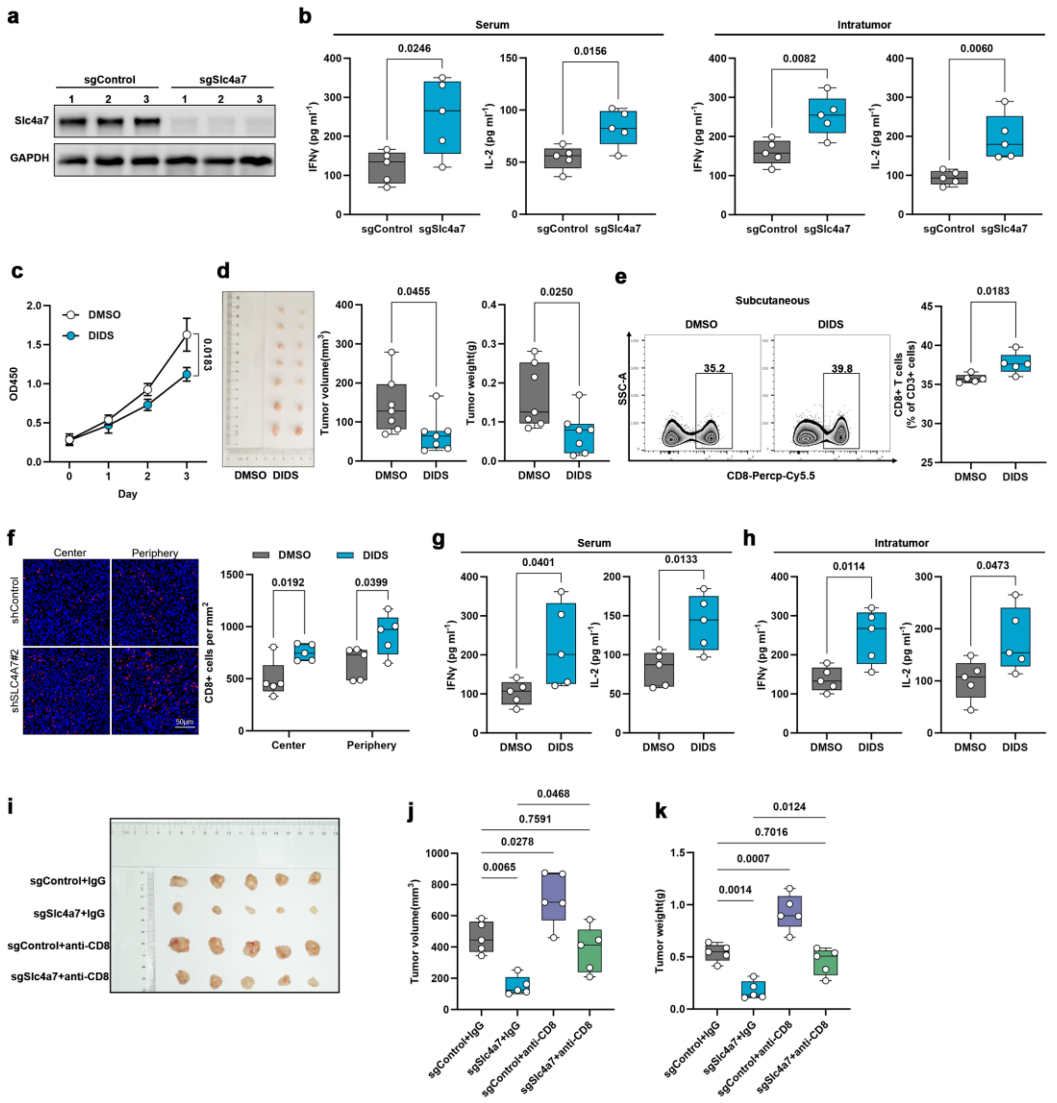

Figure S6. The anti-tumor effect of SLC4A7 knockdown is related to CD8+ T cells

a, Representative image of western blot analysis of Slc4a7 knock out in KP cells. **b,** ELISA was used to detect the concentrations of IFNγ and IL2 in the serum and tumors of mice with subcutaneous tumors (n = 5). **c,** CCK8 assay was used to detect the proliferation ability of KP cells after DIDS treatment (n = 3). **d,** Volume and weight of subcutaneous tumors in C57 mice after DIDS or DMSO treatment (n = 7). **e,** Flow cytometric analysis of the percentage of CD3+CD8+ T cells in subcutaneous tumors spleen in C57 mice after DIDS or DMSO treatment (n = 5). **f,** Representative images of infiltrating CD8+ T cells in KP subcutaneous tumors after DIDS or DMSO treatment (n = 5), bar = 50μm. **g,h,** ELISA was used to detect the concentrations of IFNγ and IL2 in

the serum and tumors of mice with subcutaneous tumors (n = 5). **i-k,** Tumor volume and weight of subcutaneous KP tumors in mice treated with anti-CD8 or IgG (n = 5). *P* value was assessed by two-tailed Student's t-test **(b-h)**, one-way ANOVA followed by the Tukey's post hoc **(j, k)**.

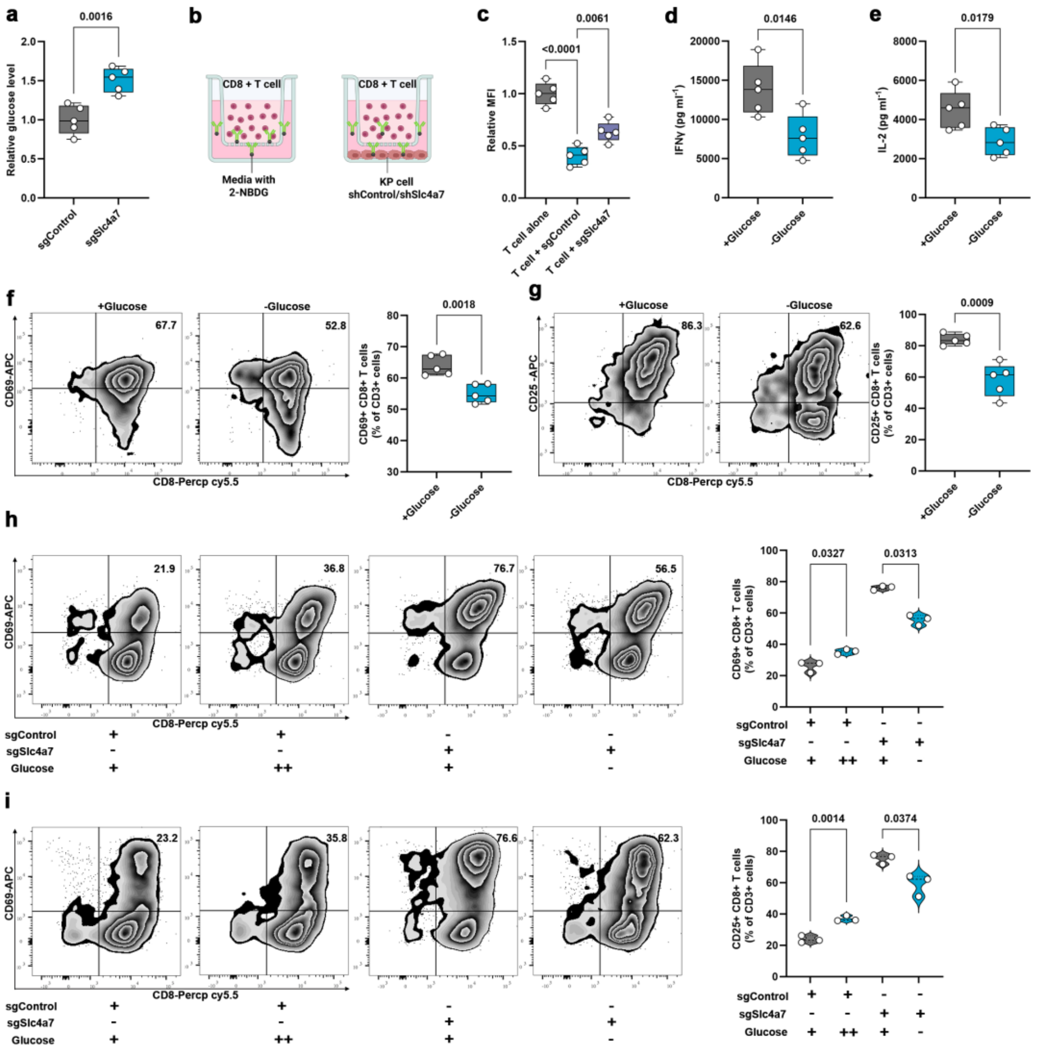

Figure S7. Glucose is essential for T cell activation

a, Glucose concentration in the supernatant of KP cells with or without Slc4a7 knockdown was analyzed using a glucose detection kit (n = 5). **b,c,** Glucose uptake in CD8+ T cells cultured with or without KP tumor cells was measured using fluorescent 2-NBDG using a fluorescence microplate reader (n = 5). **d,e,** ELISA analysis of IFNγ and IL2 secretion levels after 48 h of culture at medium with or without glucose (n = 5). **f,g,** Flow cytometry analysis of the proportion of CD69+ and CD25+ CD8+ T cells after 48 h of culture in the presence or absence of glucose (n = 5). **h,i,** Flow cytometry analysis of the effects of adding or removing glucose in KP-conditioned medium on CD8+ T cell activation after 48 hours of culture (n = 3). *P* value was assessed by two-tailed Student's t-test **(a, c-g)**, one-way ANOVA followed by the Tukey's post hoc **(h, i)**.

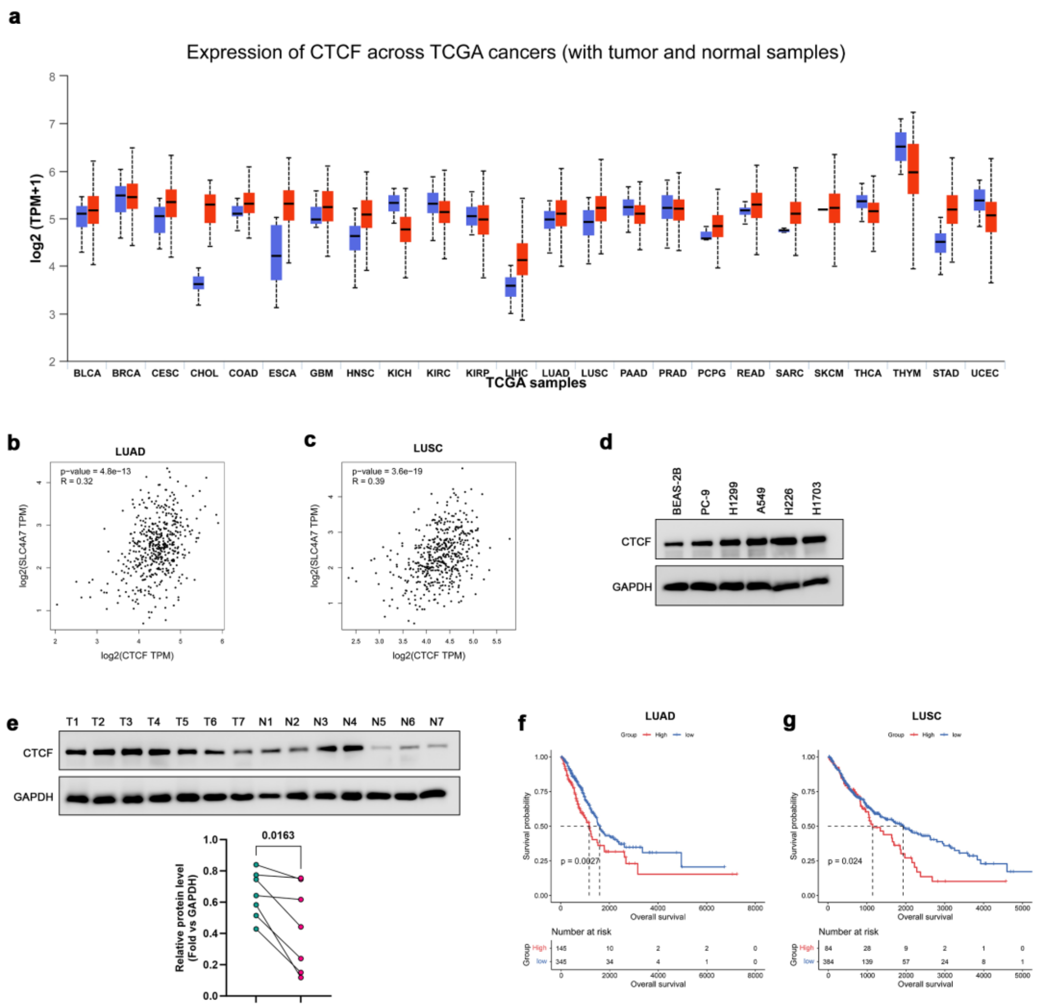

Figure S8. CTCF expression is elevated in lung cancer and is associated with clinical prognosis

a, Expression of CTCF in various tumors in the TCGA database. **b,c**, Correlation analysis between CTCF and SLC4A7 in TCGA database. **d,** Western blot analysis of SLC4A7 expression in BEAS-2B, pc-9, h1299, a549, h226, h1703 cells. **e,** Western blot analysis of SLC4A7 expression in tumor and normal tissue (n = 7) **f, g,** TCGA database shows that high expression of CTCF shortens survival time

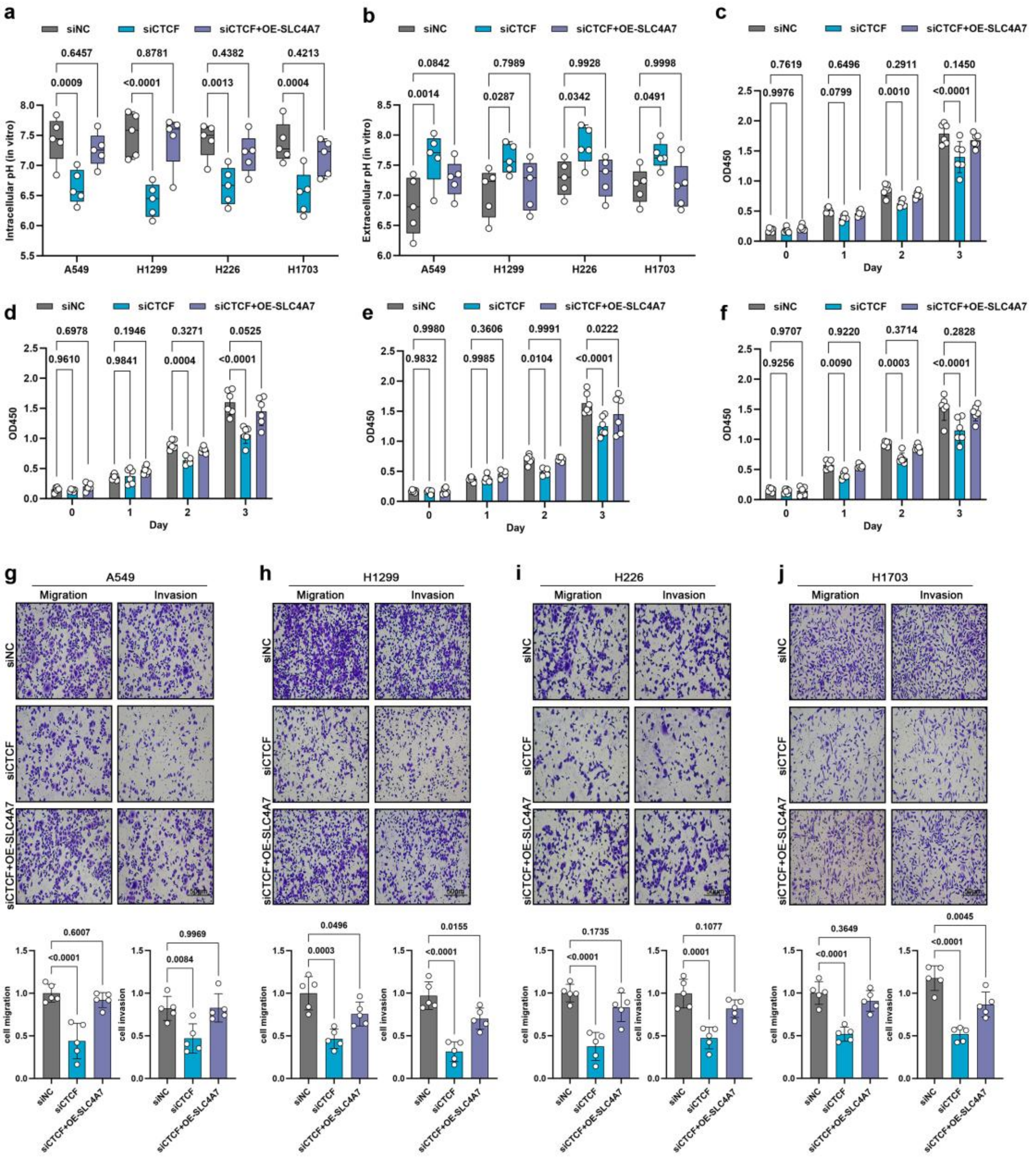

Figure S9. CTCF knockdown leads to intracellular acidification and inhibits proliferation and metastasis of NSCLC cells

a, Quantitative analysis of intracellular pH using pHrodo Red intracellular pH indicator dye in A549, H1299, H226 and H1703 cells with or without CTCF knockdown (n=5). **b,** Quantitative analysis of extracellular pH values using single-barreled H+-sensitive microelectrodes in A549, H1299, H226 and H1703 cells with or without CTCF knockdown (n=5). **c-f**, CCK8 assay was used to detect the proliferation ability of A549, H1299, H226 and H1703 cells after CTCF knockdown. (n = 6). **g-j,** Representative images and statistical charts of transwell assay of A549, H1299, H226 and H1703 cells

after CTCF knockdown (n=5), bar = 50μm. *P* value was assessed by one-way ANOVA followed by the Tukey's post hoc.

Table S1. Materials and reagents used in this article

REAGENT or RESOURCE	SOURCE	IDENTIFIER
Antibodies		
SLC4A7 Polyclonal antibody	Proteintech	Cat# 29442-1-AP; RRID: AB_3086133
SLC4A7 Polyclonal Antibody	Invitrogen	Cat# PA5-57433; RRID: AB_2647530
SLC4A7 Antibody	Affinity	Cat# DF9927; RRID: AB_2843121
CTCF (D31H2) XP Rabbit mAb	Cell Signaling Technology	Cat# 3418; RRID: AB_2086791
Monoclonal Anti-CTCF antibody produced in mouse	Sigma Aldrich	Cat# AMAB90663; AB_2665624
GPI Monoclonal antibody	Proteintech	Cat# 67178-1-Ig; RRID: AB_2882474
Hexokinase 2 Monoclonal antibody	Proteintech	Cat# 66974-1-Ig; RRID: AB_2882294
PFKFB3 Polyclonal antibody	Proteintech	Cat# 13763-1-AP; RRID: AB_2162854
Alpha Tubulin Monoclonal antibody	Proteintech	Cat# 66031-1-Ig; RRID: AB_11042766
GAPDH Monoclonal antibody	Proteintech	Cat# 60004-1-Ig; RRID: AB_2107436
E-cadherin Monoclonal antibody	Proteintech	Cat# 60335-1-Ig; RRID: AB_2881444
N-cadherin Monoclonal antibody	Proteintech	Cat# 66219-1-Ig, RRID: AB_2881610
Vimentin Monoclonal antibody	Proteintech	Cat# 60330-1-Ig; RRID: AB_2881439
Fixable Viability Stain 510	BD Biosciences	Cat# 564406; RRID: AB_2869572

Fixable Viability Stain 450	BD Biosciences	Cat#562247; RRID: AB_2869405
APC-Cy7 Rat Anti-Mouse CD45	BD Biosciences	Cat# 557659; RRID: AB_396774
APC Hamster Anti-Mouse CD3e	BD Biosciences	Cat# 553066; RRID: AB_398529
FITC anti-mouse CD3ε Antibody	Biolegend	Cat# 100306; RRID: AB_312671
R718 Rat Anti-Mouse CD8a	BD Biosciences	Cat# 566985; RRID: AB_2869989
PerCP-Cy5.5 Rat Anti-Mouse CD8a	BD Biosciences	Cat# 551162; RRID: AB_394081
FITC anti-mouse CD4 Antibody	Biolegend	Cat# 100406; RRID: AB_312691
FOXP3 Monoclonal Antibody (FJK-16s), PE	Thermo Fisher Scientific	Cat# 12-5773-82; RRID: AB_465936
F4/80 Monoclonal Antibody (BM8), PE-eFluor™ 610	Thermo Fisher Scientific	Cat# 61-4801-82; RRID:AB_2574612
FITC anti-mouse/human CD11b Antibody	Biolegend	Cat# 101206; RRID: AB_312789
PE/Cyanine7 anti-mouse CD86 Antibody	Biolegend	Cat# 105115; RRID: AB_493601
Alexa Fluor 647 Rat Anti-Mouse CD206	BD Biosciences	Cat# 565250; RRID: AB_2739133
BV421 Hamster Anti-Mouse CD11c	BD Biosciences	Cat# 562782; RRID: AB_2737789
MHC Class II (I-A/I-E) Monoclonal Antibody (M5/114.15.2), PE-Cyanine7	Thermo Fisher Scientific	Cat# 25-5321-82; RRID: AB_10870792
FITC anti-mouse CD19 Antibody	Biolegend	Cat# 115505; RRID: AB_313640
PE/Cyanine7 anti-mouse NK-1.1 Antibody	Biolegend	Cat# 108714; RRID: AB_389364
BV421 Hamster Anti-Mouse CD69	BD Biosciences	Cat# 562920; RRID: AB_2687478
CD69 Monoclonal Antibody (H1.2F3), APC	Thermo Fisher Scientific	Cat# 17-0691-80; RRID:AB_1210795
CD25 Monoclonal Antibody (PC61.5), APC	Thermo Fisher Scientific	Cat# 17-0251-81; RRID: AB_469365

CD25 Monoclonal Antibody (PC61.5), FITC	Thermo Fisher Scientific	Cat# 11-0251-80; RRID:AB_2802176
Anti-pan Cytokeratin antibody	Abcam	Cat# ab7753; RRID: AB_306047
Anti-CD8 alpha antibody	Abcam	Cat# ab237709; RRID: AB_2892677
InVivoMAb anti-mouse CD8α	BioCell	Cat# BE0004-1; RRID: AB_1107671
InVivoMAb anti-mouse PD-1 (CD279)	BioCell	Cat# BE0146; RRID: AB_10949053
InVivoMAb anti-mouse PD-L1 (B7-H1)	BioCell	Cat# BE0101; RRID: AB_10949073
Chemicals, peptides, and recombinant proteins		
Seahorse XF 1.0 M glucose solution	Aligen	Cat#103577-100
Seahorse XF 100 mM pyruvate solution	Aligent	Cat#103578-100
Seahorse XF 200 mM glutamine solution	Aligent	Cat#103579-100
3-aminopropyl phosphonate	Sigma-Aldrich	Cat#268615
HEPES (1M)	Thermo Fisher Scientific	Cat#15630080
MEM NEAA	Thermo Fisher Scientific	Cat#11140050
SODIUM PYRUVATE	Thermo Fisher Scientific	Cat#11360070
GLUTAMAX I, 100X	Thermo Fisher Scientific	Cat#35050061
Sodium bicarbonate (^{13}C, 99%)	Cambridge Isotope Laboratories	Cat#87081-58-1
Advanced DMEM/F12	Thermo Fisher Scientific	Cat# 12634010
Glutamax	Gibco	Cat# 35050

N2 SUPPLEMENT	Thermo Fisher Scientific	Cat#A1370701
B-27 SUPPLEMENT	Thermo Fisher Scientific	Cat#12587010
EGF	PeproTech	Cat#AF-100-15-500UG
Noggin	MedChemExpress	Cat#HY-P700143AF
R-spondin-1	Thermo Fisher Scientific	Cat#120-38-100UG
FGF10	MedChemExpress	Cat#HY-P7342AF
N-Acetyl-L-cysteine	MedChemExpress	Cat#HY-B0215
Y-27632	MedChemExpress	Cat#HY-10071
A83-01	MedChemExpress	Cat#HY-10432
Matrigel	Corning	Cat#354234
TrypLE Express	Thermo Fisher Scientific	Cat#12604021
Critical commercial assays		
Cell-Light EdU Apollo567 In Vitro Kit	Ribobio	Cat#C10310-1
Seahorse XF Cell Mito Stress Test Kit	Aligent	Cat#103015-100
Seahorse XF Glycolysis Stress Test Kit	Aligent	Cat#103020-100
Dynabeads Mouse T-Activator CD3/CD28 for activation of mouse T cells	Thermo Fisher Scientific	Cat#11456D
Mouse CD8+ T-Cell Isolation Kit	Selleck	Cat#B90011

Magnetic ChIP Kit	Sigma-Aldrich	Cat#17-10086
Intracellular pH Calibration Buffer Kit	Invitrogen	Cat#P35379
Deposited data		
Single cell transcriptomic	CZ CELLxGENE	https://cellxgene.cziscience.com/
RNA sequencing	This paper	
ATAC sequencing	This paper	
TCGA data RNA-seq	GDC	https://portal.gdc.cancer.gov/
Experimental models: Cell lines		
NCI-H1299	Procell	Cat#CL-0165
A549	Procell	Cat#CL-0016
NCI-H226	Procell	Cat#CL-0396
NCI-H1703	Procell	Cat#CL-0390
293T	Procell	Cat#CL-0005
KP cells	This paper	NA
Experimental models: Organisms/strains		
BALB/c-Nude	GemPharmatech	Strain NO. D000521
C57BL/6J	Charles River	NA
NOD/ShiLtJGpt-Prkdc^{em26Cd52}Il2rg^{em26Cd22}/Gpt	GemPharmatech	Strain NO. T001475
Oligonucleotides		
shSLC4A7#1 target sequence GCAATGAAACTCTAGCACAAT	This paper	NA
shSLC4A7#2 target sequence CCAGTTATTTGACCGTATAAA	This paper	NA

shSLC4A7#3 target sequence<br>CCATGAAATTGGACGATCAAT	This paper	NA
sgSlc4a7#1 target sequence<br>GTCGGCGTCACAAGCATCGA	This paper	NA
sgSlc4a7#2 target sequence<br>GAGGGCACAAACATCACCAC	This paper	NA
sgSlc4a7#3 target sequence<br>GCTAAGACACGCCATCGCAG	This paper	NA
siCTCF#1 target sequence (5’-3’)<br>GUGGAGGAGUCCGAAACUUTT<br>AAGUUUCCGACUCCUCCACTT	This paper	NA
siCTCF#2 target sequence (5′ -3′)<br>GGAAAGUGAACCCAUGAUATT<br>UAUCAUGGGUUCACUUUCCTT	This paper	NA
siCTCF#3 target sequence (5′ -3′)<br>GGGACACAUACAAGCUGAATT<br>UUCAGCUUGUAUGUGUCCCTT	This paper	NA
Software and algorithms		
FlowJo	FlowJo LLC	https://flowjovx.software.informer.com/
GraphPad Prism 10.0	GraphPad Prism	https://graphpad-prism.software.informer.com/

Table S2. Tissue microarray clinical information of all sample

Variable	Overall, N = 172[1]	High, N = 24[1]	Low, N = 56[1]	Middle, N = 92[1]	p-value[2]
**Pathological type**					0.084
LUAD	82 (48%)	12 (50%)	33 (59%)	37 (40%)	
LUSC	90 (52%)	12 (50%)	23 (41%)	55 (60%)	
**Gender**					0.036
female	35 (20%)	3 (12%)	18 (32%)	14 (15%)	
male	137 (80%)	21 (88%)	38 (68%)	78 (85%)	
**Age**					0.8
<=65	114 (66%)	15 (62%)	36 (64%)	63 (68%)	
>65	58 (34%)	9 (38%)	20 (36%)	29 (32%)	
**Tumor stage**					<0.001
Ⅰ	54 (31%)	7 (29%)	30 (54%)	17 (18%)	
Ⅱ	56 (33%)	9 (38%)	17 (30%)	30 (33%)	
Ⅲ	60 (35%)	8 (33%)	9 (16%)	43 (47%)	
IV	2 (1.2%)	0 (0%)	0 (0%)	2 (2.2%)	
**Distant metastasis**					>0.9
negative	168 (98%)	24 (100%)	55 (98%)	89 (97%)	
positive	4 (2.3%)	0 (0%)	1 (1.8%)	3 (3.3%)	
**Lymph node metastasis**					0.008
negative	92 (53%)	13 (54%)	39 (70%)	40 (43%)	
positive	80 (47%)	11 (46%)	17 (30%)	52 (57%)	
**EGFR**					0.3

Table S2. Tissue microarray clinical information of all sample

Variable	Overall, N = 172[1]	High, N = 24[1]	Low, N = 56[1]	Middle, N = 92[1]	p-value[2]
negative	52 (30%)	9 (38%)	21 (38%)	22 (24%)	
positive	10 (5.8%)	2 (8.3%)	2 (3.6%)	6 (6.5%)	
unknown	110 (64%)	13 (54%)	33 (59%)	64 (70%)	
**ALK**					0.2
negative	51 (30%)	8 (33%)	17 (30%)	26 (28%)	
positive	14 (8.1%)	4 (17%)	6 (11%)	4 (4.3%)	
unknown	107 (62%)	12 (50%)	33 (59%)	62 (67%)	
**Status**					<0.001
alive	76 (44%)	6 (25%)	37 (66%)	33 (36%)	
dead	96 (56%)	18 (75%)	19 (34%)	59 (64%)	

[1]n (%)

[2]Pearson's Chi-squared test; Fisher's exact test

Table S3. Tissue microarray clinical information of LUAD

Variable	Overall, N = 82[1]	High, N = 12[1]	Low, N = 33[1]	Middle, N = 37[1]	p-value[2]
**Gender**					0.071
female	32 (39%)	3 (25%)	18 (55%)	11 (30%)	
male	50 (61%)	9 (75%)	15 (45%)	26 (70%)	
**Age**					0.8
<=65	57 (70%)	9 (75%)	24 (73%)	24 (65%)	
>65	25 (30%)	3 (25%)	9 (27%)	13 (35%)	
**Tumor stage**					0.084
Ⅰ	32 (39%)	4 (33%)	18 (55%)	10 (27%)	
Ⅱ	23 (28%)	5 (42%)	9 (27%)	9 (24%)	
Ⅲ	26 (32%)	3 (25%)	6 (18%)	17 (46%)	
IV	1 (1.2%)	0 (0%)	0 (0%)	1 (2.7%)	
**Distant metastasis**					>0.9
negative	80 (98%)	12 (100%)	32 (97%)	36 (97%)	
positive	2 (2.4%)	0 (0%)	1 (3.0%)	1 (2.7%)	
**Lymph node metastasis**					0.041
negative	46 (56%)	6 (50%)	24 (73%)	16 (43%)	
positive	36 (44%)	6 (50%)	9 (27%)	21 (57%)	
**EGFR**					0.4
negative	52 (63%)	9 (75%)	21 (64%)	22 (59%)	
positive	10 (12%)	2 (17%)	2 (6.1%)	6 (16%)	
unknown	20 (24%)	1 (8.3%)	10 (30%)	9 (24%)	
**ALK**					0.086
negative	51 (62%)	8 (67%)	17 (52%)	26 (70%)	
positive	14 (17%)	4 (33%)	6 (18%)	4 (11%)	

Table S3. Tissue microarray clinical information of LUAD

Variable	Overall, N = 82[1]	High, N = 12[1]	Low, N = 33[1]	Middle, N = 37[1]	p-value[2]
unknown	17 (21%)	0 (0%)	10 (30%)	7 (19%)	
**Status**					0.036
alive	33 (40%)	3 (25%)	19 (58%)	11 (30%)	
dead	49 (60%)	9 (75%)	14 (42%)	26 (70%)	

[1]n (%)

[2]Fisher's exact test; Pearson's Chi-squared test

Table S4. Tissue microarray clinical information of LUAD

Variable	Overall, N = 90[1]	High, N = 12[1]	Low, N = 23[1]	Middle, N = 55[1]	p-value[2]
**Gender**					0.7
female	3 (3.3%)	0 (0%)	0 (0%)	3 (5.5%)	
male	87 (97%)	12 (100%)	23 (100%)	52 (95%)	
**Age**					0.14
<=65	57 (63%)	6 (50%)	12 (52%)	39 (71%)	
>65	33 (37%)	6 (50%)	11 (48%)	16 (29%)	
**Tumor stage**					0.006
I	22 (24%)	3 (25%)	12 (52%)	7 (13%)	
II	33 (37%)	4 (33%)	8 (35%)	21 (38%)	
III	34 (38%)	5 (42%)	3 (13%)	26 (47%)	
IV	1 (1.1%)	0 (0%)	0 (0%)	1 (1.8%)	
**Distant metastasis**					>0.9
negative	88 (98%)	12 (100%)	23 (100%)	53 (96%)	
positive	2 (2.2%)	0 (0%)	0 (0%)	2 (3.6%)	
**Lymph node metastasis**					0.2
negative	46 (51%)	7 (58%)	15 (65%)	24 (44%)	
positive	44 (49%)	5 (42%)	8 (35%)	31 (56%)	
**EGFR**					
unknown	90 (100%)	12 (100%)	23 (100%)	55 (100%)	
**ALK**					
unknown	90 (100%)	12 (100%)	23 (100%)	55 (100%)	
**Status**					0.002
alive	43 (48%)	3 (25%)	18 (78%)	22 (40%)	
dead	47 (52%)	9 (75%)	5 (22%)	33 (60%)	

Table S4. Tissue microarray clinical information of LUAD

Variable	Overall, N = 90[1]	High, N = 12[1]	Low, N = 23[1]	Middle, N = 55[1]	p-value[2]

[1]n (%)

[2]Fisher's exact test; Pearson's Chi-squared test

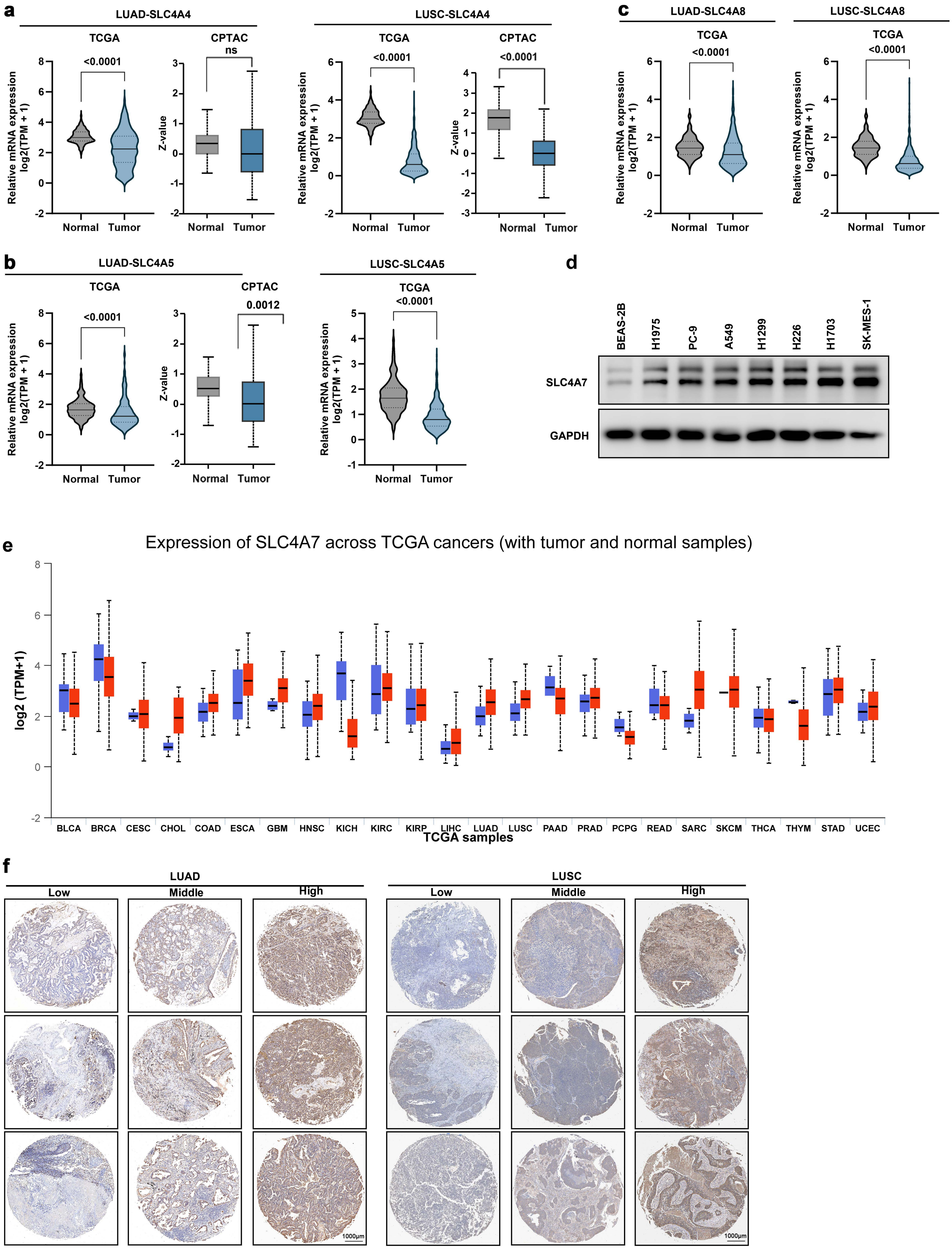
a
LUAD-SLC4A4
TCGA
CPTAC
ns
<0.0001
LUSC-SLC4A4
Relative mRNA expression log2(TPM + 1)
Z-value
Normal
Tumor
c
LUAD-SLC4A8
LUSC-SLC4A8
b
LUAD-SLC4A5
0.0012
LUSC-SLC4A5
d
BEAS-2B
H1975
PC-9
A549
H1299
H226
H1703
SK-MES-1
SLC4A7
GAPDH
e
Expression of SLC4A7 across TCGA cancers (with tumor and normal samples)
log2 (TPM+1)
BLCA
BRCA
CESC
CHOL
COAD
ESCA
GBM
HNSC
KICH
KIRC
KIRP
LIHC
LUAD
LUSC
PAAD
PRAD
PCPG
READ
SARC
SKCM
THCA
THYM
STAD
UCEC
TCGA samples
f
LUAD
LUSC
Low
Middle
High
1000μm

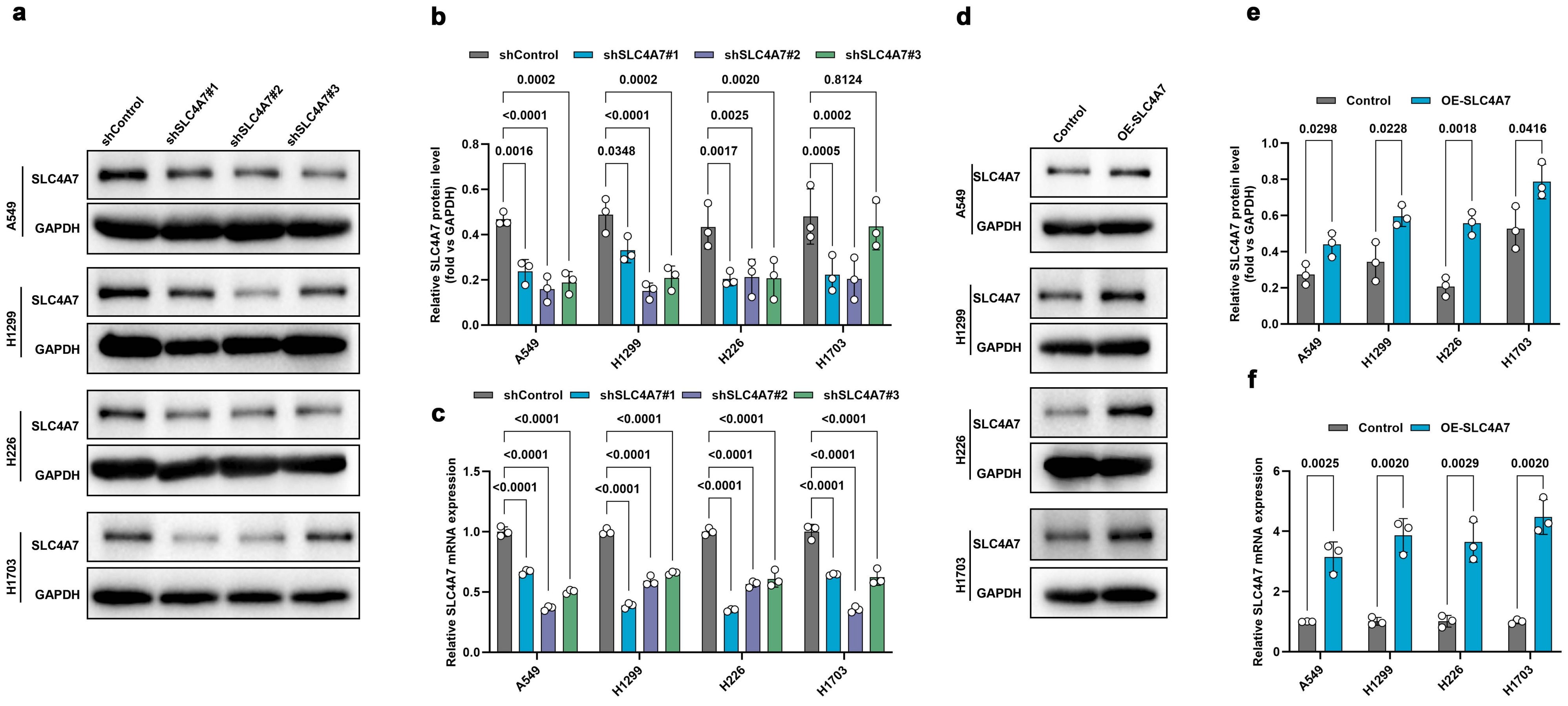

a
shControl
shSLC4A7#1
shSLC4A7#2
shSLC4A7#3
A549
H1299
H226
H1703
SLC4A7
GAPDH
b
shControl
shSLC4A7#1
shSLC4A7#2
shSLC4A7#3
Relative SLC4A7 protein level (fold vs GAPDH)
0.0
0.2
0.4
0.6
0.8
0.0002
<0.0001
0.0016
0.0002
<0.0001
0.0348
0.0020
0.0025
0.0017
0.8124
0.0002
0.0005
A549
H1299
H226
H1703
c
shControl
shSLC4A7#1
shSLC4A7#2
shSLC4A7#3
Relative SLC4A7 mRNA expression
0.0
0.5
1.0
1.5
<0.0001
d
Control
OE-SLC4A7
A549
H1299
H226
H1703
SLC4A7
GAPDH
e
Control
OE-SLC4A7
Relative SLC4A7 protein level (fold vs GAPDH)
0.0
0.2
0.4
0.6
0.8
1.0
0.0298
0.0228
0.0018
0.0416
A549
H1299
H226
H1703
f
Control
OE-SLC4A7
Relative SLC4A7 mRNA expression
0
2
4
6
0.0025
0.0020
0.0029
0.0020
A549
H1299
H226
H1703

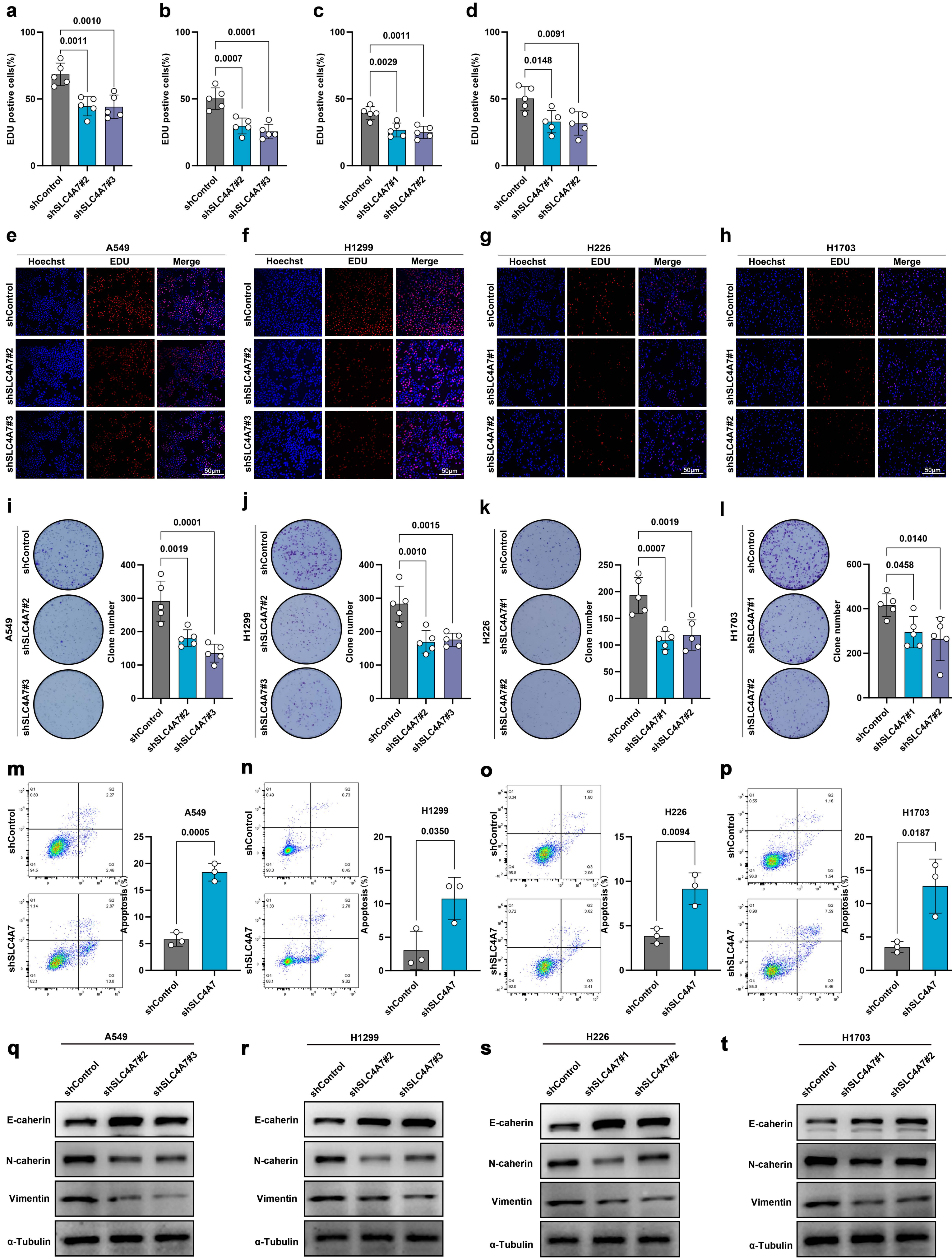
a
b
c
d
EDU postive cells(%)
0.0010
0.0011
0.0001
0.0007
0.0011
0.0029
0.0091
0.0148
shControl
shSLC4A7#1
shSLC4A7#2
shSLC4A7#3
e
A549
f
H1299
g
H226
h
H1703
Hoechst
EDU
Merge
50μm
i
j
k
l
A549
H1299
H226
H1703
Clone number
0.0001
0.0019
0.0015
0.0010
0.0019
0.0007
0.0140
0.0458
m
n
o
p
Apoptosis (%)
0.0005
0.0350
0.0094
0.0187
shSLC4A7
q
r
s
t
E-caherin
N-caherin
Vimentin
α-Tubulin

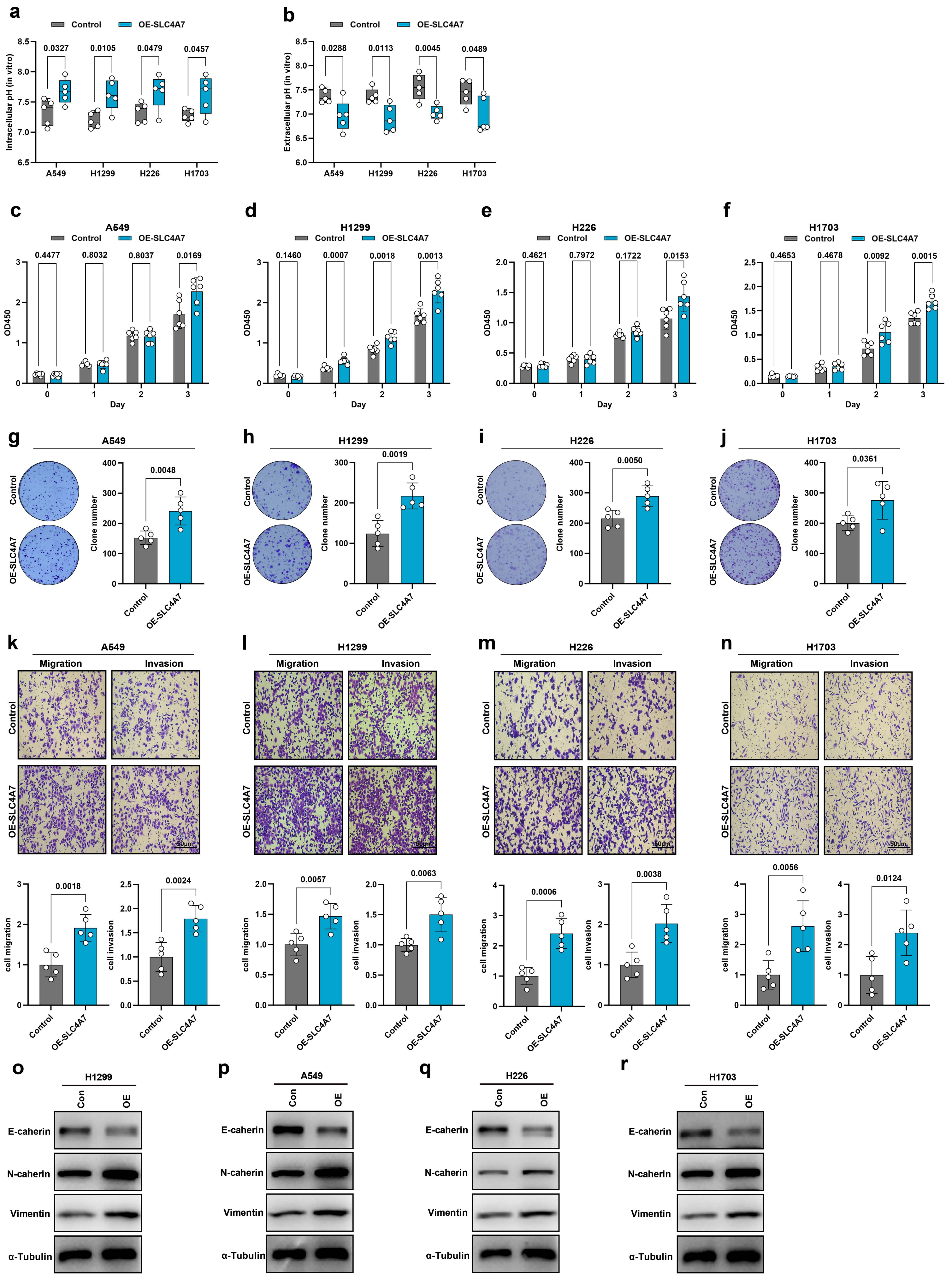

a
Control
OE-SLC4A7
Intracellular pH (in vitro)
0.0327
0.0105
0.0479
0.0457
A549
H1299
H226
H1703
b
Extracellular pH (in vitro)
0.0288
0.0113
0.0045
0.0489
c
A549
0.4477
0.8032
0.8037
0.0169
OD450
Day
d
H1299
0.1460
0.0007
0.0018
0.0013
e
H226
0.4621
0.7972
0.1722
0.0153
f
H1703
0.4653
0.4678
0.0092
0.0015
g
0.0048
Clone number
h
0.0019
i
0.0050
j
0.0361
k
Migration
Invasion
50μm
0.0018
0.0024
cell migration
cell invasion
l
0.0057
0.0063
m
0.0006
0.0038
n
0.0056
0.0124
o
Con
OE
E-caherin
N-caherin
Vimentin
α-Tubulin
p
q
r

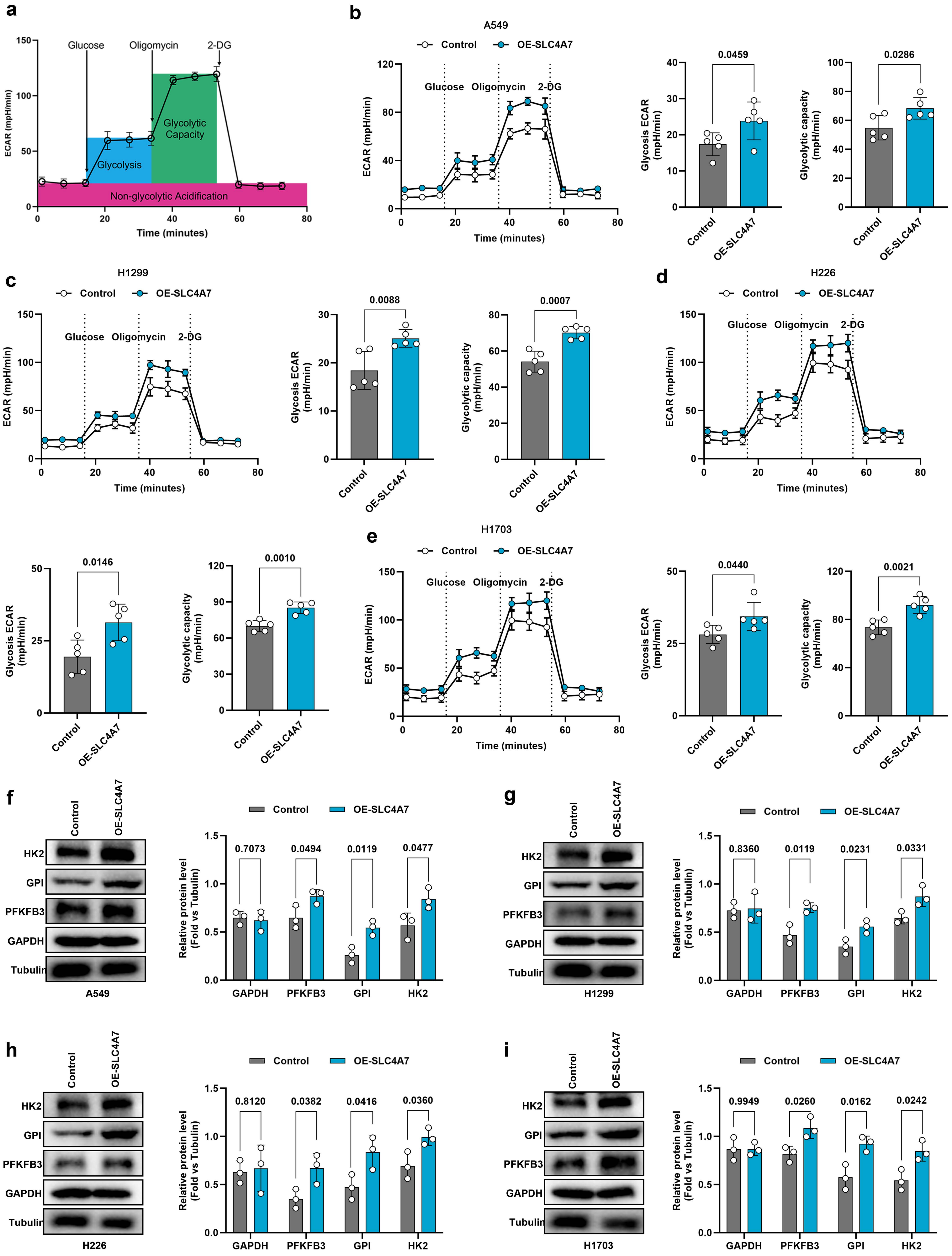
a
Glucose
Oligomycin
2-DG
Glycolysis
Glycolytic Capacity
Non-glycolytic Acidification
ECAR (mpH/min)
Time (minutes)
b
A549
Control
OE-SLC4A7
ECAR (mpH/min)
Time (minutes)
Glycosis ECAR (mpH/min)
Glycolytic capacity (mpH/min)
0.0459
0.0286
c
H1299
0.0088
0.0007
d
H226
0.0146
0.0010
e
H1703
0.0440
0.0021
f
HK2
GPI
PFKFB3
GAPDH
Tubulin
A549
Relative protein level (Fold vs Tubulin)
0.7073
0.0494
0.0119
0.0477
g
H1299
0.8360
0.0119
0.0231
0.0331
h
H226
0.8120
0.0382
0.0416
0.0360
i
H1703
0.9949
0.0260
0.0162
0.0242

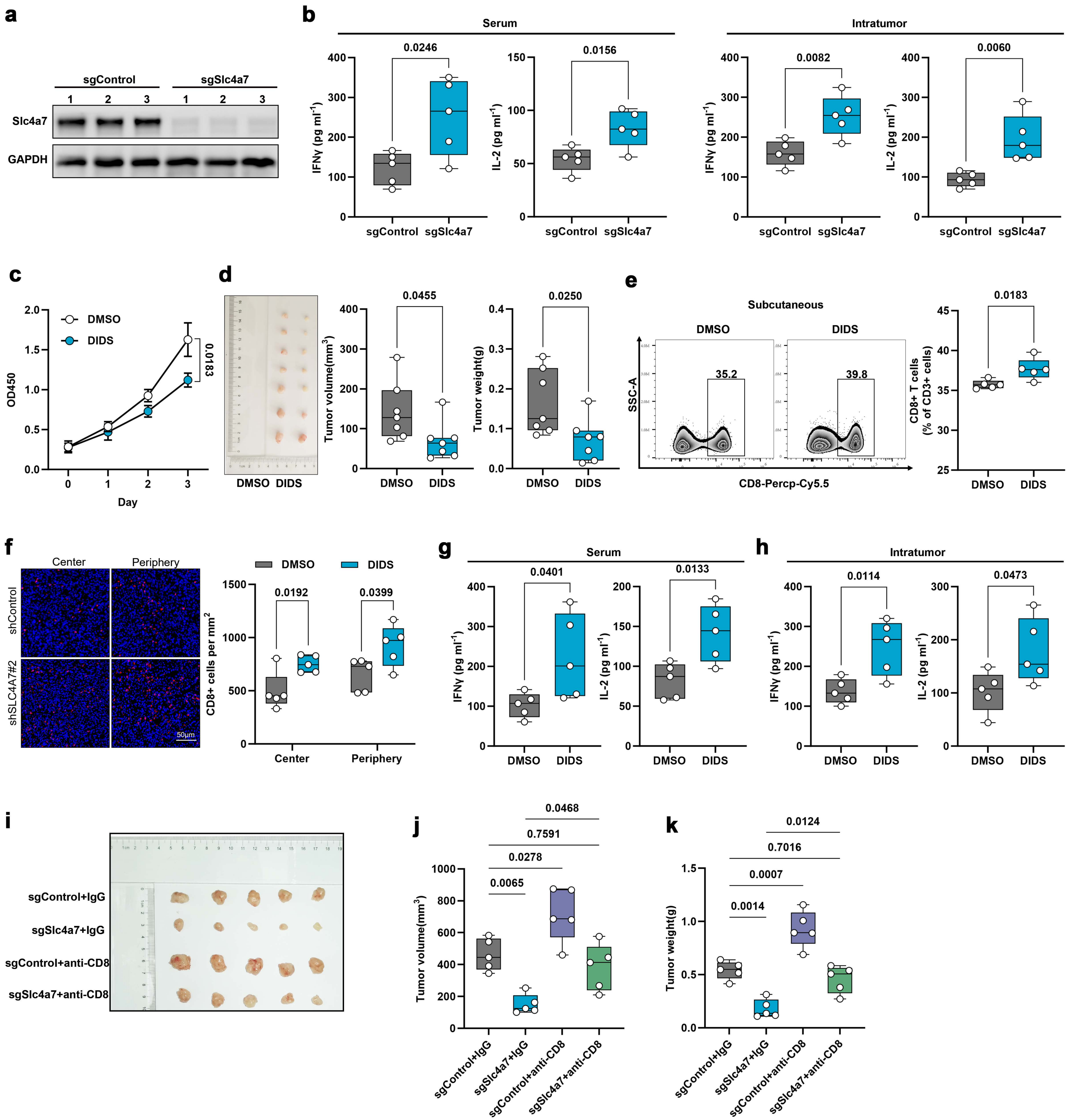

a
sgControl
sgSlc4a7
1 2 3 1 2 3
Slc4a7
GAPDH
b
Serum
Intratumor
0.0246
0.0156
0.0082
0.0060
IFNγ (pg ml-1)
IL-2 (pg ml-1)
sgControl sgSlc4a7
c
DMSO
DIDS
OD450
Day
0.0183
d
DMSO DIDS
0.0455
0.0250
Tumor volume(mm3)
Tumor weight(g)
e
Subcutaneous
DMSO
DIDS
35.2
39.8
SSC-A
CD8-Percp-Cy5.5
0.0183
CD8+ T cells (% of CD3+ cells)
f
Center
Periphery
shControl
shSLC4A7#2
50μm
DMSO
DIDS
0.0192
0.0399
CD8+ cells per mm2
g
Serum
0.0401
0.0133
h
Intratumor
0.0114
0.0473
i
sgControl+IgG
sgSlc4a7+IgG
sgControl+anti-CD8
sgSlc4a7+anti-CD8
j
0.0468
0.7591
0.0278
0.0065
Tumor volume(mm3)
k
0.0124
0.7016
0.0007
0.0014
Tumor weight(g)

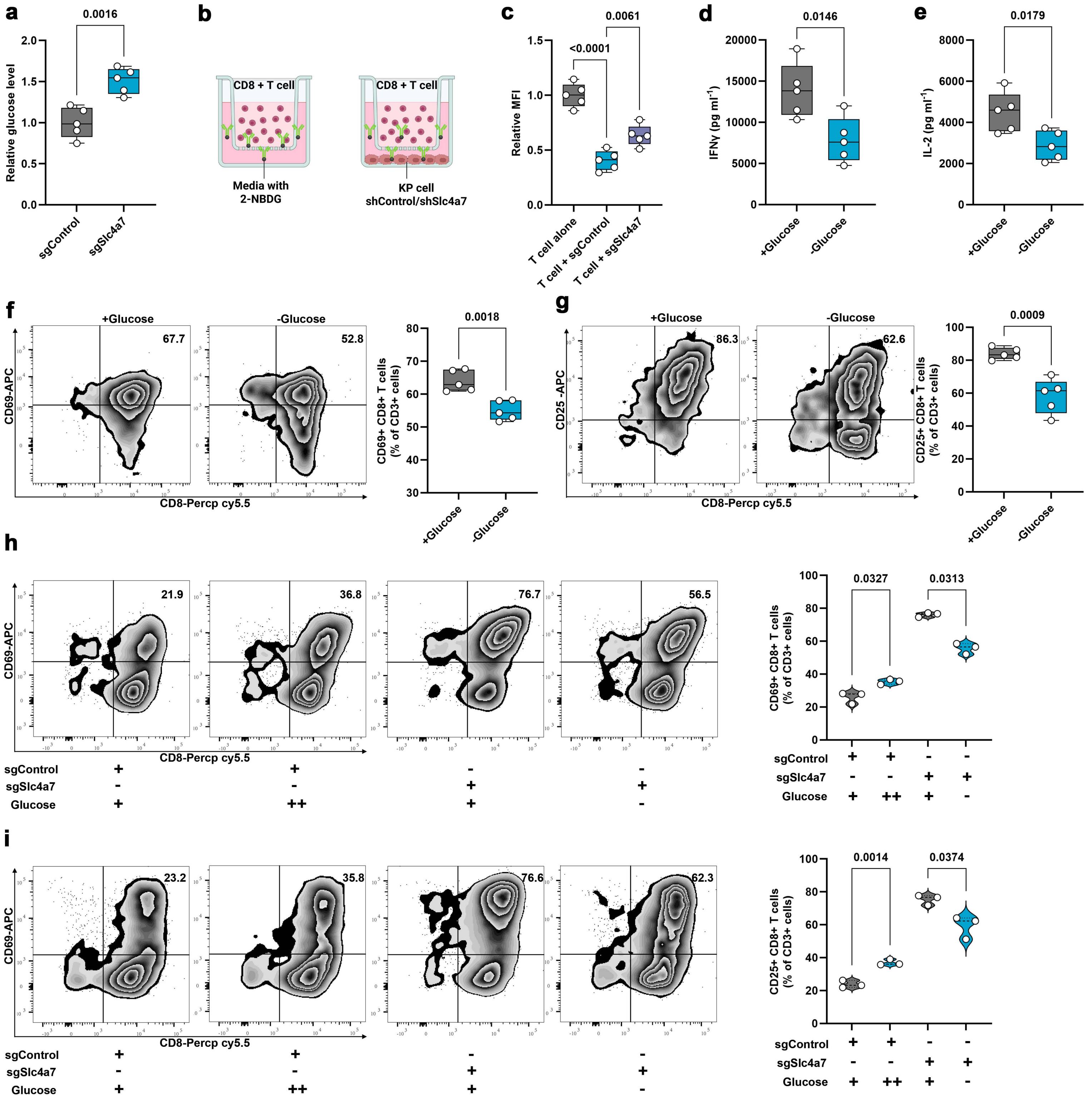

a
0.0016
Relative glucose level
sgControl
sgSlc4a7
b
CD8 + T cell
Media with 2-NBDG
KP cell shControl/shSlc4a7
c
0.0061
<0.0001
Relative MFI
T cell alone
T cell + sgControl
T cell + sgSlc4a7
d
0.0146
IFNγ (pg ml-1)
+Glucose
-Glucose
e
0.0179
IL-2 (pg ml-1)
+Glucose
-Glucose
f
+Glucose
-Glucose
67.7
52.8
CD69-APC
CD8-Percp cy5.5
0.0018
CD69+ CD8+ T cells (% of CD3+ cells)
g
+Glucose
-Glucose
86.3
62.6
CD25 -APC
CD8-Percp cy5.5
0.0009
CD25+ CD8+ T cells (% of CD3+ cells)
h
21.9
36.8
76.7
56.5
CD69-APC
CD8-Percp cy5.5
sgControl
sgSlc4a7
Glucose
0.0327
0.0313
CD69+ CD8+ T cells (% of CD3+ cells)
i
23.2
35.8
76.6
62.3
CD69-APC
CD8-Percp cy5.5
0.0014
0.0374
CD25+ CD8+ T cells (% of CD3+ cells)

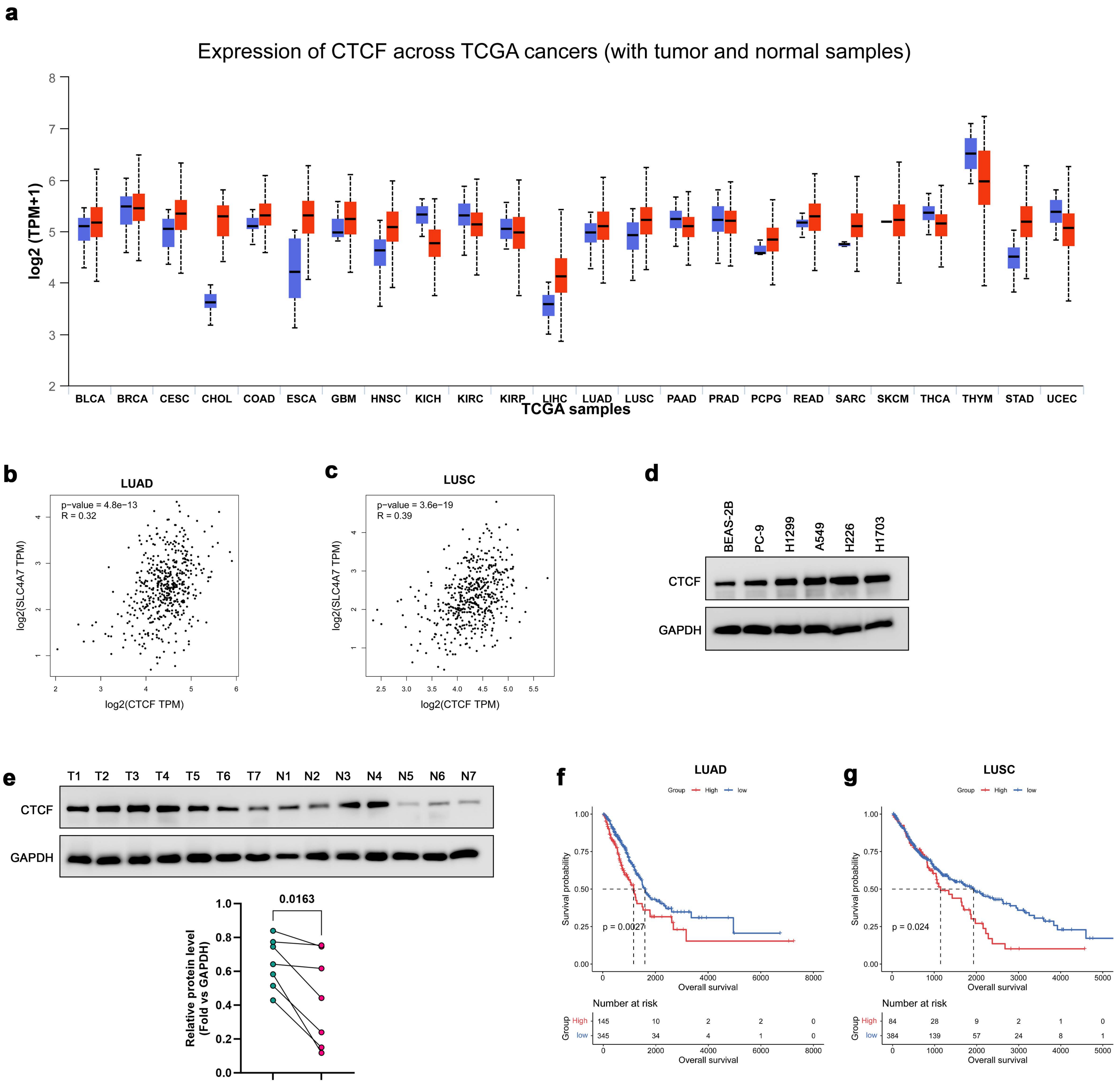
a
Expression of CTCF across TCGA cancers (with tumor and normal samples)
log2 (TPM+1)
TCGA samples
BLCA BRCA CESC CHOL COAD ESCA GBM HNSC KICH KIRC KIRP LIHC LUAD LUSC PAAD PRAD PCPG READ SARC SKCM THCA THYM STAD UCEC
b
LUAD
p−value = 4.8e−13
R = 0.32
log2(SLC4A7 TPM)
log2(CTCF TPM)
c
LUSC
p−value = 3.6e−19
R = 0.39
log2(SLC4A7 TPM)
log2(CTCF TPM)
d
BEAS-2B
PC-9
H1299
A549
H226
H1703
CTCF
GAPDH
e
T1 T2 T3 T4 T5 T6 T7 N1 N2 N3 N4 N5 N6 N7
CTCF
GAPDH
0.0163
Relative protein level (Fold vs GAPDH)
f
LUAD
Group High low
Survival probability
p = 0.0027
Overall survival
Number at risk
Group
High 145 10 2 2 0
low 345 34 4 1 0
Overall survival
g
LUSC
Group High low
Survival probability
p = 0.024
Overall survival
Number at risk
Group
High 84 28 9 2 1 0
low 384 139 57 24 8 1
Overall survival

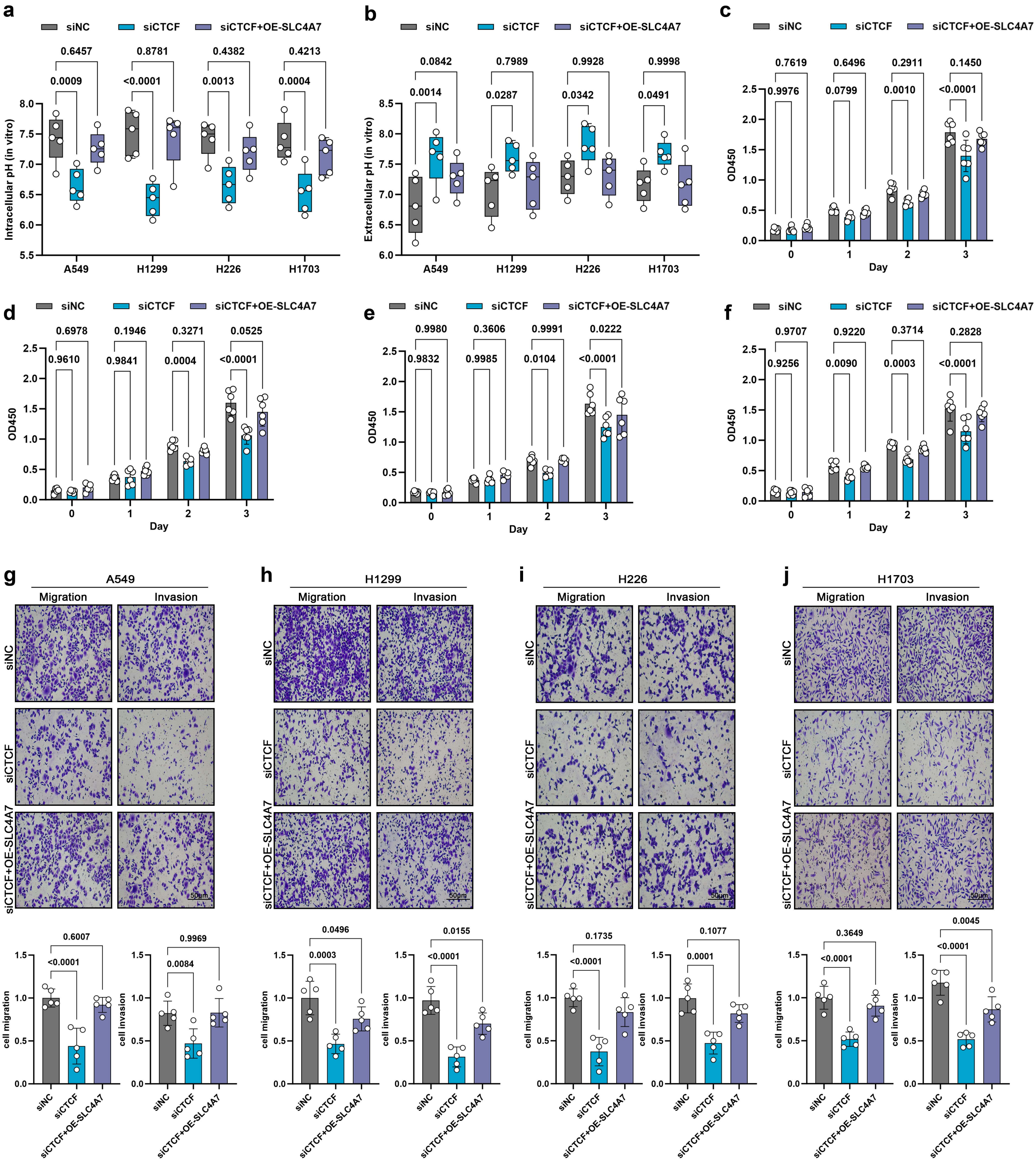
a
siNC
siCTCF
siCTCF+OE-SLC4A7
0.6457
0.8781
0.4382
0.4213
0.0009
<0.0001
0.0013
0.0004
Intracellular pH (in vitro)
A549
H1299
H226
H1703
b
0.0842
0.7989
0.9928
0.9998
0.0014
0.0287
0.0342
0.0491
Extracellular pH (in vitro)
c
0.7619
0.6496
0.2911
0.1450
0.9976
0.0799
0.0010
<0.0001
OD450
Day
d
0.6978
0.1946
0.3271
0.0525
0.9610
0.9841
0.0004
<0.0001
e
0.9980
0.3606
0.9991
0.0222
0.9832
0.9985
0.0104
<0.0001
f
0.9707
0.9220
0.3714
0.2828
0.9256
0.0090
0.0003
<0.0001
g
A549
Migration
Invasion
h
H1299
i
H226
j
H1703
50μm
0.6007
<0.0001
0.9969
0.0084
0.0496
0.0003
0.0155
<0.0001
0.1735
<0.0001
0.1077
0.0001
0.3649
<0.0001
0.0045
<0.0001
cell migration
cell invasion
